# The invasiveness of human cervical cancer associated to the function of Na_V_1.6 channels is mediated by MMP-2 activity

**DOI:** 10.1038/s41598-018-31364-y

**Published:** 2018-08-29

**Authors:** Osbaldo Lopez-Charcas, Ana Maria Espinosa, Ana Alfaro, Zazil Herrera-Carrillo, Belen Ernestina Ramirez-Cordero, Pedro Cortes-Reynosa, Eduardo Perez Salazar, Jaime Berumen, Juan Carlos Gomora

**Affiliations:** 1 Departamento de Neuropatología Molecular, Instituto de Fisiología Celular, Universidad Nacional Autónoma de México, Ciudad de México, 04510 Mexico; 2 Unidad de Medicina Genómica, Facultad de Medicina, Universidad Nacional Autónoma de México/Hospital General de México, Ciudad de México, 06720 Mexico; 3 Departamento de Biología Celular, Cinvestav-IPN, Av. IPN # 2508, San Pedro Zacatenco, Ciudad de México, 07360 Mexico

## Abstract

Voltage-gated sodium (Na_V_) channels have been related with cell migration and invasiveness in human cancers. We previously reported the contribution of Na_V_1.6 channels activity with the invasion capacity of cervical cancer (CeCa) positive to Human Papilloma Virus type 16 (HPV16), which accounts for 50% of all CeCa cases. Here, we show that Na_V_1.6 gene (*SCN8A*) overexpression is a general characteristic of CeCa, regardless of the HPV type. In contrast, no differences were observed in Na_V_1.6 channel expression between samples of non-cancerous and cervical intraepithelial neoplasia. Additionally, we found that CeCa cell lines, C33A, SiHa, CaSki and HeLa, express mainly the splice variant of *SCN8A* that lacks exon 18, shown to encode for an intracellularly localized Na_V_1.6 channel, whereas the full-length adult form was present in CeCa biopsies. Correlatively, patch-clamp experiments showed no evidence of whole-cell sodium currents (I_Na_) in CeCa cell lines. Heterologous expression of full-length Na_V_1.6 isoform in C33A cells produced I_Na_, which were sufficient to significantly increase invasion capacity and matrix metalloproteinase type 2 (MMP-2) activity. These data suggest that upregulation of Na_V_1.6 channel expression occurs when cervical epithelium have been transformed into cancer cells, and that Na_V_1.6-mediated invasiveness of CeCa cells involves MMP-2 activity. Thus, our findings support the notion about using Na_V_ channels as therapeutic targets against cancer metastasis.

## Introduction

Cervical cancer (CeCa) is the second most frequent female cancer worldwide with more than half a million new cases every year; and about 250,000 deaths annually, which locates CeCa as the third leading cause of cancer-related deaths in females in developing countries. The human papillomavirus (HPV) is present in virtually all CeCa patients and it is considered the main risk factor for developing this carcinoma. Fifteen HPV genotypes have been classified as ‘high-risk’ due to their oncogenic potential and they are associated with most CeCa patients^1^. HPV type 16 (HPV16) is the most frequent accounting for more than 50% of CeCa cases, followed by HPV18 (17%) and others (25%); altogether high-risk HPV types are responsible for more than 95% of all CeCa cases^1^. Around fifteen percent of CeCa patients are diagnosed as metastatic cervical cancer (MCC) which has a poor survival prognosis^2,3^. Particularly, matrix metalloproteinases (MMPs) have been associated with cervical cancer progression as in other human cancers^4–6^. Commercial vaccines against HPV16 and HPV18 have been very effective to prevent infection of cervical epithelium, also in preventing the development of high-grade cervical intraepithelial neoplasia associated with these HPV types. However, these vaccines are limited to offer protection only for a few of the fifteen high-risk HPV types and it is still unknown whether the immune response will remain unchanged until the age of peak incidence for CeCa. In addition, predictions of global incidence and mortality for CeCa display an increase if vaccinated women are not included in early screening programs for CeCa^2^. Therefore, to develop new strategies for CeCa early detection and new therapeutic approaches for metastatic cervical cancer remains as an urgent goal.

Voltage-gated sodium (Na_V_) channels are protein complexes formed by a large pore-forming α-subunit and smaller auxiliary β-subunit. Since their first description, Na_V_s have been canonically related to the generation and propagation of action potentials in excitable cells^7^. However, more recently several studies have shown that Na_V_s are functionally expressed in several epithelial cancers (breast, cervix, colon, gastric, lung, prostate, ovarium), as well as in other cancer types (glioma and leukemia), while they are not or are poorly expressed in the cognate non-cancerous tissue^8,9^. The abnormal expression of Na_V_s in human malign cells has been mainly associated with the invasiveness and cancer progression^10–17^.

Mechanistic issues about participation of Na_V_s on invasive properties of cancer cells has been widely studied in human breast cancer^18–21^ and more recently in gastric cancer^10^. The pore-forming Na_V_1.5 subunit is expressed in highly aggressive human breast cancer cells but it has not been associated with the triggering of action potentials. Instead, it enhances extracellular matrix (ECM) degradation by increasing the activity of the Na^+^/H^+^ exchanger 1 (NHE-1)^18,19^, promoting a consecutive activation of extracellular acidic cysteine cathepsins, and by modifying F-actin polymerization via Src kinase activity to acquire a cellular invasive morphology which altogether promote invadopodial activity and cell invasiveness^18–20^. Additionally, the loss of *SCN4B* in human breast cancer cells, gene that encodes for the Na_V_β4 subunit of VGSCs, promotes the acquisition of an amoeboid-mesenchymal hybrid phenotype associated with metastases, while its overexpression reduces cancer cell invasiveness^22^, demonstrating new non-canonical functions for the auxiliary Na_V_β subunits in addition to those shown for the pore-forming α-subunits of Na_V_s. In addition, a recent study showed that Na_V_1.7 channels encoded by the *SCN9A* gene is abundantly expressed in human gastric cancer where its activity induced an increase in NHE-1 expression, proliferation, invasion, and expression of the oncoprotein *metastasis-associated in colon cancer-1* (MACC1)^10^.

Another sodium channel, the Na_V_1.6 isoform (encoded by the *SCN8A* gene) has been found to be expressed exclusively in macrophages derived from human monocytic leukemia and cancer cells from human melanoma but exclusively in intracellular vesicles. The activity of this sodium channel contributes to the cellular invasion through its effects on podosome and invadopodia formation via a mechanism involving intracellular movement of sodium and calcium ions as well as F-actin cytoskeletal remodeling in these cells^23^.

We have previously reported the functional expression of Na_V_s in cervical cancer (CeCa) biopsies and primary cultures positives to HPV16. Among all Na_V_s, the Na_V_1.6 isoform is specifically overexpressed and has a direct contribution to the invasion capacity of these cancer cells. In addition, Na_V_1.6 protein showed a distinct subcellular distribution in cancer versus non-cancer cells, suggesting a cancer-associated relocation of these sodium channels to the plasma membrane^24,25^. However, whether these findings about the relevance of Na_V_1.6 channels in CeCa positive to HPV16 are conserved in the other 50% of total cases of CeCa, remains as a pending issue. Also, the mechanism involved in the Na_V_1.6-mediated cervical cancer cell invasiveness has not been addressed previously. In this study, we investigated the expression of *SCN8A* gene in biopsies representing almost all subtypes of human CeCa. We also explored the expression of *SCN8A* and Na_V_1.6 protein in the neoplasia-carcinoma sequence of human cervical tissue, using samples from non-cancerous cervix, low- and high-grade cervical intraepithelial neoplasia and invasive cervical cancer. In addition, by using a heterologous expression system we obtain the first insights into the mechanism underlying the cell invasion driven by Na_V_1.6 channels in CeCa, which involves the specific secretion and activity of MMP-2. The findings of this work improve our understanding of CeCa invasion mechanisms and provide the experimental support for the consideration of Na_V_1.6 channels as molecular targets for cervical cancer therapy.

## Results

### The overexpression of *SCN8A* is a distinctive characteristic of invasive cervical cancer

**Figure 1:**
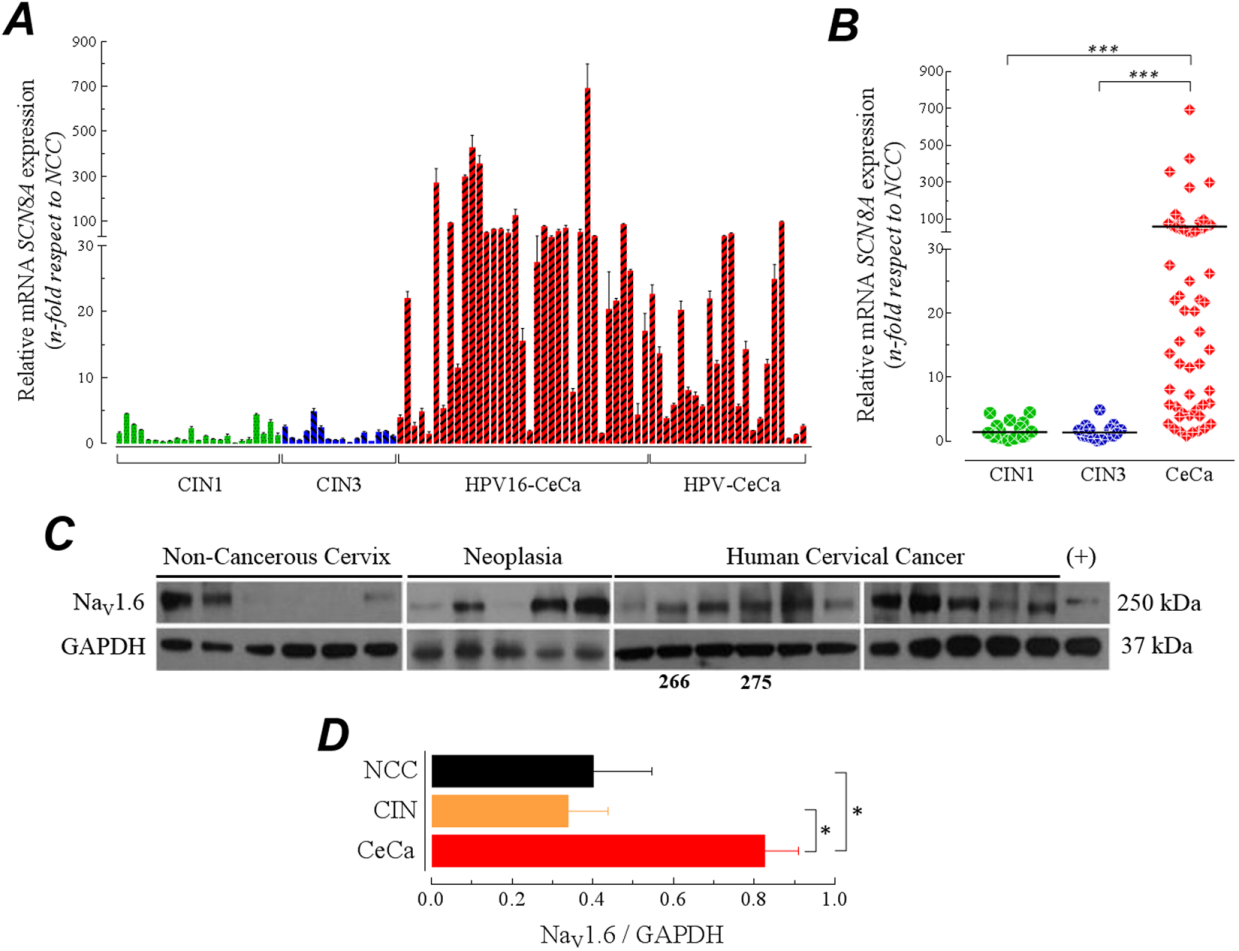
Exploration of *SCN8A* expression in the neoplasia-carcinoma sequence of human cervical tissue. (**A**) Expression levels of *SCN8A* gene in low-grade (CIN1, *n* = 23) and high-grade cervical intraepithelial neoplasia (CIN2/3, *n* = 16), as well as in invasive cervical cancer positive to HPV16 (HPV16-CeCa, *n* = 35) and invasive cervical cancer positive to other oncogenic HPV types (HPV-CeCa, *n* = 22), *versus* non-cancerous cervix (NCC, *n* = 20). Bars show the average fold-change ratio (2^−ΔΔCt^) of *SCN8A* gene for individual samples of each group. (**B**) Scattering plot of fold-change values for each group of samples. Horizontal black lines represent the fold-change mean values. *SCN8A* gene was significantly upregulated in cervical cancer tissues (Mann-Whitney U test, *P* < 0.0001). (**C**) Western blot analysis of Na_V_1.6 channel expression in total protein extracts from NCC, CIN and CeCa samples. Total protein extracts from HEK293 cells stably expressing Na_V_1.6 channels were used as positive control. Representative results of four independent experiments. Numbers below blots correspond to sample number. Samples 266 and 275 are those that were present in all western blots experiments shown in the present work. (**D**) Relative expression of Na_V_1.6 protein in cervical tissue samples. Blots were quantified by densitometry and normalized to that of GAPDH for NCC (*n* = 12), CIN (*n* = 10) and CeCa (*n* = 29). Asterisks indicates *P* < 0.05 with a Student’s t-test.

The Na_V_1.6 channels are highly expressed in both central and peripheral nervous system particularly at nodes of Ranvier, synapses and dendrites where they play an important role in generating action potentials for the high speed propagation of electrical signals^26^. Consequently, the loss of Na_V_1.6 function cause severe motor and cognitive disorders^27,28^. On the other hand, there are several evidences that show the expression of Na_V_1.6 channels in normal non-excitable and cancer cells in which they actively participate in physiological^29–33^ and pathophysiological^11,12,23,34^ processes, respectively. We have previously demonstrated that the *SCN8A* gene, which encodes the Na_V_1.6 sodium channel, was forty-times more abundant in CeCa positive to HPV16 than in non-cancerous cervix^25^. Therefore, we investigated whether this abnormal expression of *SCN8A* gene was a shared feature for CeCa biopsies positive to other major oncogenic HPV types. Total RNA was isolated from human non-cancerous cervix (NCC; *n* = 19); low-grade cervical intraepithelial neoplasia (CIN1; *n* = 23), high-grade cervical intraepithelial neoplasia (CIN2/3; *n* = 16) and invasive cervical cancer positive to the main oncogenic HPV types: 16, 18, 31, 45, 51, 52, 58, 59 and 68 (CeCa; *n* = 57) for the experiments of quantitative PCR. Expression of 18S gene was assessed in all samples and this was used as a reference gene for the quantitative analysis (Supplementary Fig. S1). The expression analysis indicated an upregulation of *SCN8A* gene in the invasive CeCa samples regardless of the oncogenic HPV type, however such change was not observed in low- and high-grade cervical intraepithelial neoplasia (Fig. 1A,B and Supplementary Fig. S2), supporting the idea that *SCN8A* overexpression is associated with oncogenic transformation. In addition, there was no association between expression levels of *SCN8A* and cervical cancer staging, nor with the cellular origin of CeCa; *i.e*., glandular or squamous cells (Supplementary Figs S2 and S3). In order to test the potential use of the abnormal expression of *SCN8A* gene as a molecular marker for invasive cervical cancer we performed a Receiver Operating Characteristic (ROC) curve analysis using the qPCR data from non-cancerous and CeCa samples. ROC analysis indicated that is possible to discriminate between non-cancerous samples from those of invasive cervical cancer with a higher sensitivity (~98%; Supplementary Fig. S4), than the 55% reported for the pap smear test^35^, meaning that *SCN8A* gene levels could be more effective in identifying true positive cases that the typical pap smear test.

**Figure 2:**
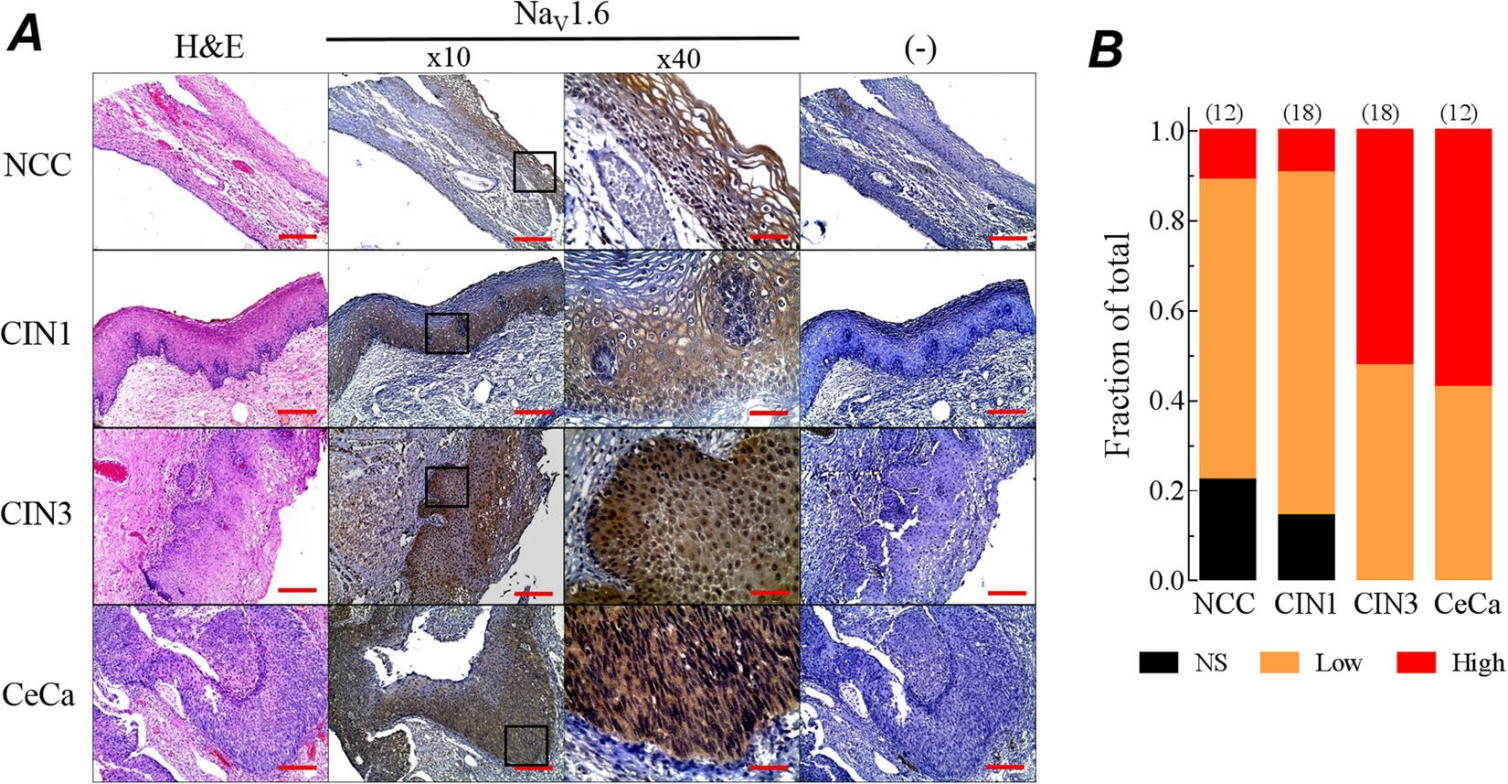
Immunohistochemical analysis of Na_V_1.6 channels in human cervical tissue. (**A**) Representative images of non-cancerous cervix (NCC), low-grade cervical intraepithelial neoplasia (CIN1), high-grade cervical intraepithelial neoplasia (CIN3) and invasive cervical cancer (CeCa), showing H&E staining (left column); immunohistochemical detection of Na_V_1.6 channel at ×10 and ×40 amplifications (middle columns); and negative controls (right column), where a non-specific IgG primary antibody was used in parallel slides. Scale bars represent 100 μm, and 300 μm for ×40 amplifications, respectively. (**B**) Fraction of total staining for each sample analyzed. Immunoreactivity intensity was evaluated as no signal (NS, black), Low (orange) and high (red) Na_V_1.6 staining in NCC, CIN1, CIN3 and CeCa tissue samples. The number of analyzed slides is indicated in parenthesis at the top of each column.

Because correlation between expression levels of mRNA and protein in biological systems are notoriously poor^36^, we decided to explore semi-quantitatively the expression level of Na_V_1.6 protein in human cervical tissue. Total protein extracts from non-cancerous cervix, cervical intraepithelial neoplasia and cervical cancer samples were used for the immunoblotting experiments. We found the Na_V_1.6 protein in the three groups, but it was almost twice more abundant in the cervical cancer group compared to non-cancerous or neoplasia groups. Furthermore, the protein of Na_V_1.6 channels was found in one hundred percent of the cervical cancer samples while only fifty- and eighty-percent of the non-cancerous cervix and neoplasia samples, respectively, showed the signal for the Na_V_1.6 protein (Fig. 1C,D). These western-blotting results were also confirmed by conventional immunohistochemical analysis of Na_V_1.6 channels in human cervix tissue. Several homemade microarray of tissue containing biopsies from non-cancerous, low- and high-grade neoplasia and cancer (Fig. 2A) from human cervix were used to determine the Na_V_1.6 protein immunoreactivity. Most of cervical tissue samples shown immunoreaction for Na_V_1.6 protein, which clearly increases as a function of tissue transformation. Levels of Na_V_1.6 protein expression were similar in low-grade cervical intraepithelial neoplasia (CIN1) and non-cancerous cervix (NCC) (Fig. 2A, top two rows), but they were substantially lower compared to high-grade cervical intraepithelial neoplasia (CIN3) and CeCa (Fig. 2A, bottom two rows), suggesting that the up-regulation of Na_V_1.6 protein occurs when the human cervical cells have been transformed and the tissue shows histological invasive characteristics. Importantly, Na_V_1.6 protein appear to be completely distributed along the cervical cancer cells contrasting with a well located plasma membrane distribution in non-cancerous cervical cells (Fig. 2A, top row, ×10 and ×40 images), as previously reported for our group^25^. On the contrary, no staining was observed in the consecutive tissue slides incubated with an irrelevant primary antibody (Fig. 2, far right column). These results show that the over-expression of the Na_V_1.6 protein is a common characteristic in the invasive CeCa, but not for earlier stages of this carcinoma. In addition, immunochemistry results suggest a change in the subcellular localization of the channel protein as observed previously in CeCa biopsies positive to HPV-16^25^.

### Most human cervical cancer cell lines do not show sodium currents in whole-cell patch-clamp recordings

**Figure 3:**
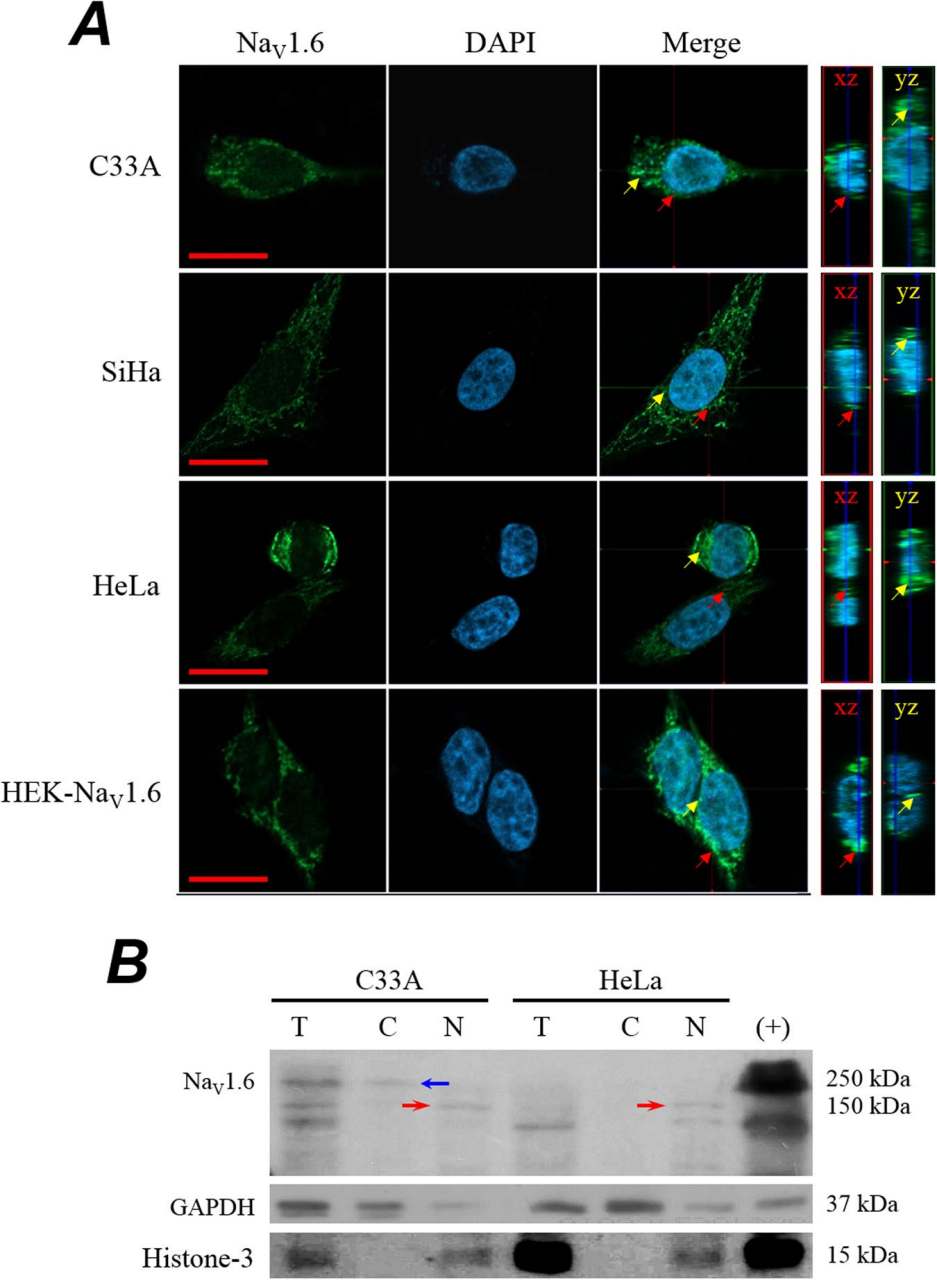
Expression of Na_V_1.6 channels in human cervical cancer cell lines. (**A**) Immunofluorescence confocal microscopy analysis of Na_V_1.6 channels expression in C33A, SiHa, HeLa, and HEK293 cells stably expressing Na_V_1.6 channels (HEK-Nav1.6; positive control). Cervical cancer cells were incubated with an antibody against Na_V_1.6 protein followed by a staining with FITC-coupled secondary antibody. DAPI reagent was used for nucleus staining. Image acquisition was performed each 0.33 µm in a total thickness of 6.5 µm. Confocal sections were merged and 3D-reconstructions were performed from Z-planes for each region of interest. Orthogonal projections from *xz* and *yz* planes of confocal images show positive signal for Na_V_1.6 protein (*far right panel*), indicated by red arrows in *xz* and yellow arrows in *yz* planes, respectively. Scale bar, 10 µm. (**B**) Western blot analysis of Na_V_1.6 channel protein in total (T), cytoplasmic (C) and nuclear (N) protein extracts from human cervical cancer cell lines. Histone-3 and GAPDH proteins were used to demonstrate the enriching of subcellular fractions. Representative blot of three independent experiments. Notice that a ~150 kDa anti-Na_V_1.6 reactive protein (red arrows) was found in nuclear fraction from cancer cells. The full-length ~250 kDa Na_V_1.6 protein was only observed in total and cytoplasmic protein extract from C33A cells.

In order to gain insights into the mechanism involve in the Na_V_1.6-mediated invasiveness of CeCa^25^, we turn to the use of commercially available human CeCa cell lines. In contrast with the functional expression of Na_V_ channels in cancer cells derived from human cervical tumors^24,25^, by using the whole-cell patch-clamp technique, we did not find voltage-gated sodium currents in the widely used HeLa (positive to HPV18) or SiHa (positive to HPV16) cells (*n* > 50 cells). We also explored three additional CeCa cell lines, CaSki (positive to HPV16), DotC2 (negative to HPV) and ME-180 cells (positive to HPV68), and under our experimental conditions, none of them show any evidence of voltage-gated sodium currents. An exception was the C33A cell line (negative to HPV), where a small percentage of cells (around 11%) express tiny, but clearly distinguishable, sodium currents (Supplementary Fig. S5). It is worth noting that blocking of this current with 1 µM TTX did not modify the basal invasiveness of C33 A cells, nor that of SiHa and HeLa cells (Supplementary Fig. S6). Regardless of the huge discrepancy between the primary cancer cells isolated from biopsies and the commercial CeCa cell lines, several possibilities could explain the lack of sodium currents in those latter cells; here we will take in consideration the following three: (1) the mRNA is not being translated into channel protein; (2) it is translated but not translocate to the plasmatic membrane but to another intracellular compartment; and (3) the channel is at the plasma membrane but is not functional. Therefore, we performed Real Time PCR (qPCR), immunofluorescence confocal microscopy, immunoblotting and conventional PCR to explore the expression of Na_V_1.6 channels in human cervical cancer cell lines. First, qPCR experiments clearly shown that CeCa cell lines express similar levels of Na_V_1.6 channel messenger (*SCN8A* gene) to those found in some CeCa tumor biopsies (Supplementary Fig. 1). In addition, the results also indicated different levels of messenger expression among these cells, as C33 A and SiHa cells contain 32-fold more copies of the Na_V_1.6 messenger than HeLa cells. Then we look for evidences of the Na_V_1.6 channel protein in this CeCa cell lines. Results of confocal microscopy show a less intense and more diffuse Na_V_1.6-positive signal in cervical cancer cells compared with positive control (Fig. 3A, right column). In all cell lines, the Na_V_1.6 channel signal was stronger in plasma membrane vicinity; interestingly, a cytosolic signal was observed mostly for CeCa cell lines (Fig. 3A, Merge column). This was better observed in three-dimensional reconstruction of images acquired in the z-plane, which suggest an intracellular distribution of Na_V_1.6 protein in cervical cancer cells lines, but not for the positive control which showed an intense signal close to the plasma membrane (Fig. 3A. Merge column). Our second approach, to obtain evidences about cellular distribution of Na_V_1.6 protein in cervical cancer cell lines, was carrying out western blot experiments on total, cytoplasmic and nuclear fractions of cell lysates. The results showed a full-length Na_V_1.6 protein (~250 kDa) in total lysate and cytoplasmic fraction of C33A cells, but the corresponding band was absent in all fractions of HeLa cells (Fig. 3B). Interestingly, a band of ~150 kDa was detected in the enriched nuclear fraction of C33A and HeLa cells (Fig. 3B, red head arrows). The enrichment of nuclear and cytoplasmic cellular fractions was evidenced by identification of Histone-3 and GAPDH proteins, respectively. These results agree with the observation of tiny sodium currents in C33A cells, displaying full length of Na_V_1.6 channel; and with the lack of those in HeLa cells, which only exhibit the smaller band of around 150 kDa. This band could be accounting for a truncated isoform of Na_V_1.6^37^.

### Differential expression of *SCN8A* splice variants between biopsies and cell lines of cervical cancer

**Figure 4:**
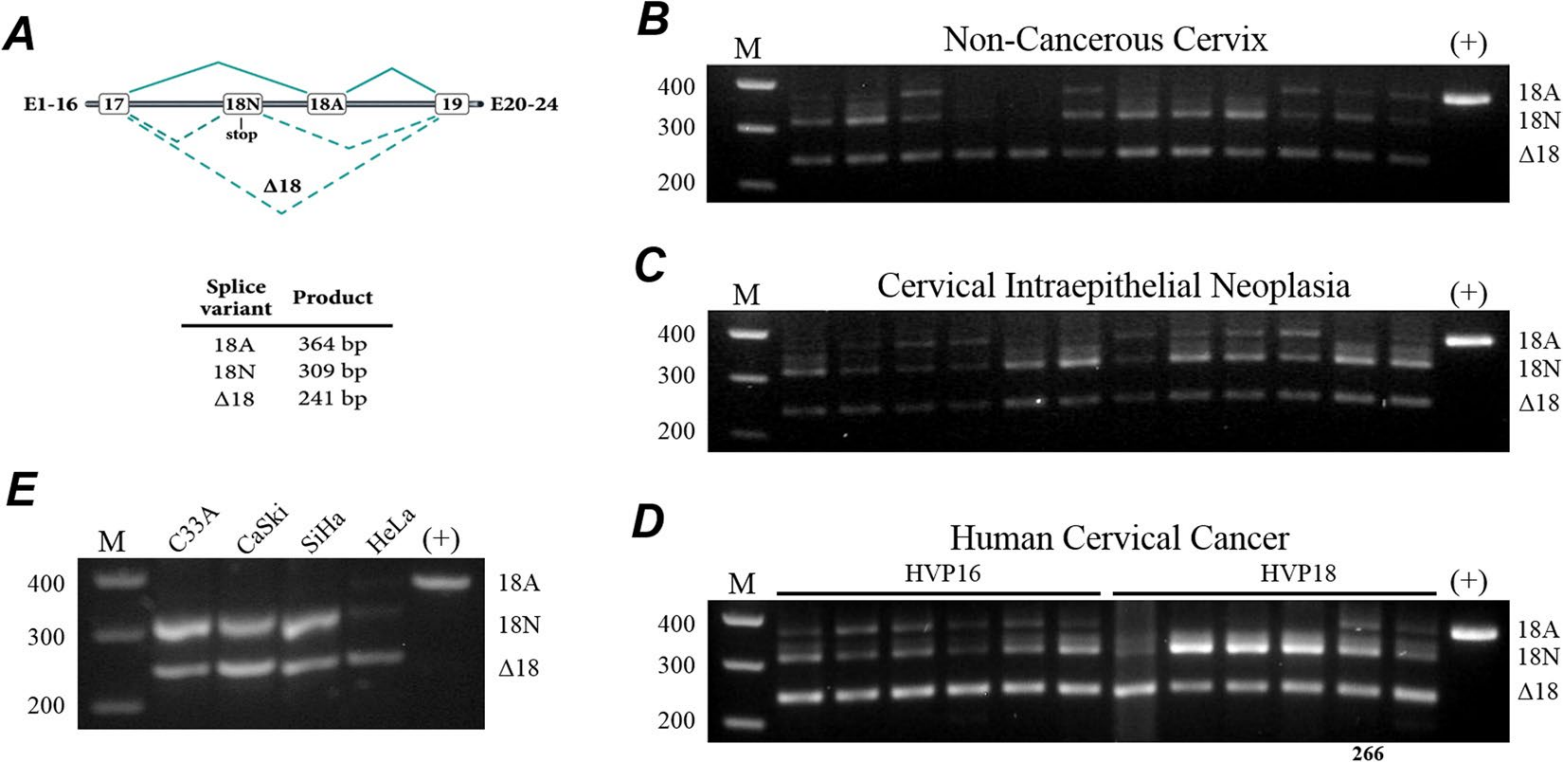
Alternative splicing of *SCN8A* exon 18 in the neoplasia-carcinoma sequence of human cervical tissue. (**A**) Alternative splicing of *SCN8A* Exon 18. Expanded genomic structure of exons 17 to 19. Exon 18 N contains an in frame stop codon. Splice variants generated by alternative splicing of Exon 18 are indicated with the PCR product length expected by using primers located in Exons 17 and 19 (see Methods). (**B**–**E**) End-point PCR electrophoresis results for *SCN8A* exon 18 variants expressed in non-cancerous cervix, cervical intraepithelial neoplasia, invasive cervical cancer, and cervical cancer cell lines, respectively. HEK-Nav1.6 cells was used as positive control for the adult splice form of *SCN8A* (18A; far-right line). A 100-bp molecular weight marker was used as reference (far-left line). The *SCN8A* variants generated for alternative splicing of exon 18: 18A, 18N and Δ18, were identified in the indicated group of samples. Identity of the *SCN8A* splice forms was confirmed by automated sequencing. The *SCN8A* splice forms were relatively more abundant in human cervical cancer samples, more clearly for the Δ18 variant; whereas the adult (18A) variant was practically absent in CeCa cell lines. From the two samples (266 and 275) that were present in all western blots experiments, only mRNA from sample 266 was available for performing these PCR analysis.

The processing of Na_V_1.6 gene (*SCN8A*) generates three different variants due to the alternative splicing of Exon 18 (Fig. 4A), which in turn encodes transmembrane segments S3 and S4 in Domain III of Na_V_1.6 channel^37^. The full length is the adult variant (18A), which is functionally express in plasma membrane of most neurons^38^; the neonatal transcript (18N) predicts a truncated two-domain protein of about 1282 amino acids (∼145 kDa), which do not express sodium currents^39,40^. The third variant is designated Δ18, this transcript maintains an open reading frame but lacks sequence encoding the S3 and S4 segments of Domain III. The expression of this variant seems to be limited to intracellular vesicular compartments regulating cellular invasion of macrophages and melanoma cells^23^. Therefore, the band of ∼150 kDa detected in HeLa cells would correspond to the 18 N variant of Na_V_1.6 channel, and could explain the absence of sodium currents in the plasma membrane of CeCa cells. In order to further explore this possibility, the presence of these exon 18 variants was assessed by standard PCR protocols in non-cancerous, neoplasia and cancer biopsies from human cervical tissue as well as in cervical cancer cell lines. Primers flanking exon 18 of the *SCN8A* gene were used to amplify the 18 A, 18 N and Δ18 products with predicted sizes of 367, 314 and 244 pair bases (pb), respectively. Again, HEK293 cells stably expressing the adult form (18A) of the human *SCN8A* gene were used as positive control for these experiments. The results show that both 18N and Δ18 splice products of *SCN8A* were consistently amplified from all groups of samples studied here, although these products were clearly more abundant in samples of cervical cancer regardless of the oncogenic HPV type (Fig. 4B–D). On the other hand, the adult variant (18A) was found in 58% of non-cancerous cervical samples (Fig. 4B), 75% of neoplasia samples (Fig. 4C), and in 100% of the cervical cancer samples positive to HPV16 (Fig. 4D, left). Interestingly, only 33% of cervical cancer samples positive to HPV18 showed the 18 A splice variant (Fig. 4D, right). Additionally, cervical cancer cell lines express predominantly the 18N and Δ18 splice variants; whereas the adult variant was practically absent in C33A, SiHa CaSki and HeLa cells (Fig. 4E). In summary, the lack of detectable voltage-gated sodium currents in CeCa cell lines, in particular those generated by the Na_V_1.6 channel could be due to the preferential expression of variants 18N and Δ18 (not functional at the plasma membrane), over the adult variant (18A), the only one that generates sodium currents at the plasma membrane.

### Heterologous expression of Na_V_1.6 increases invasiveness of CeCa cell lines

**Figure 5:**
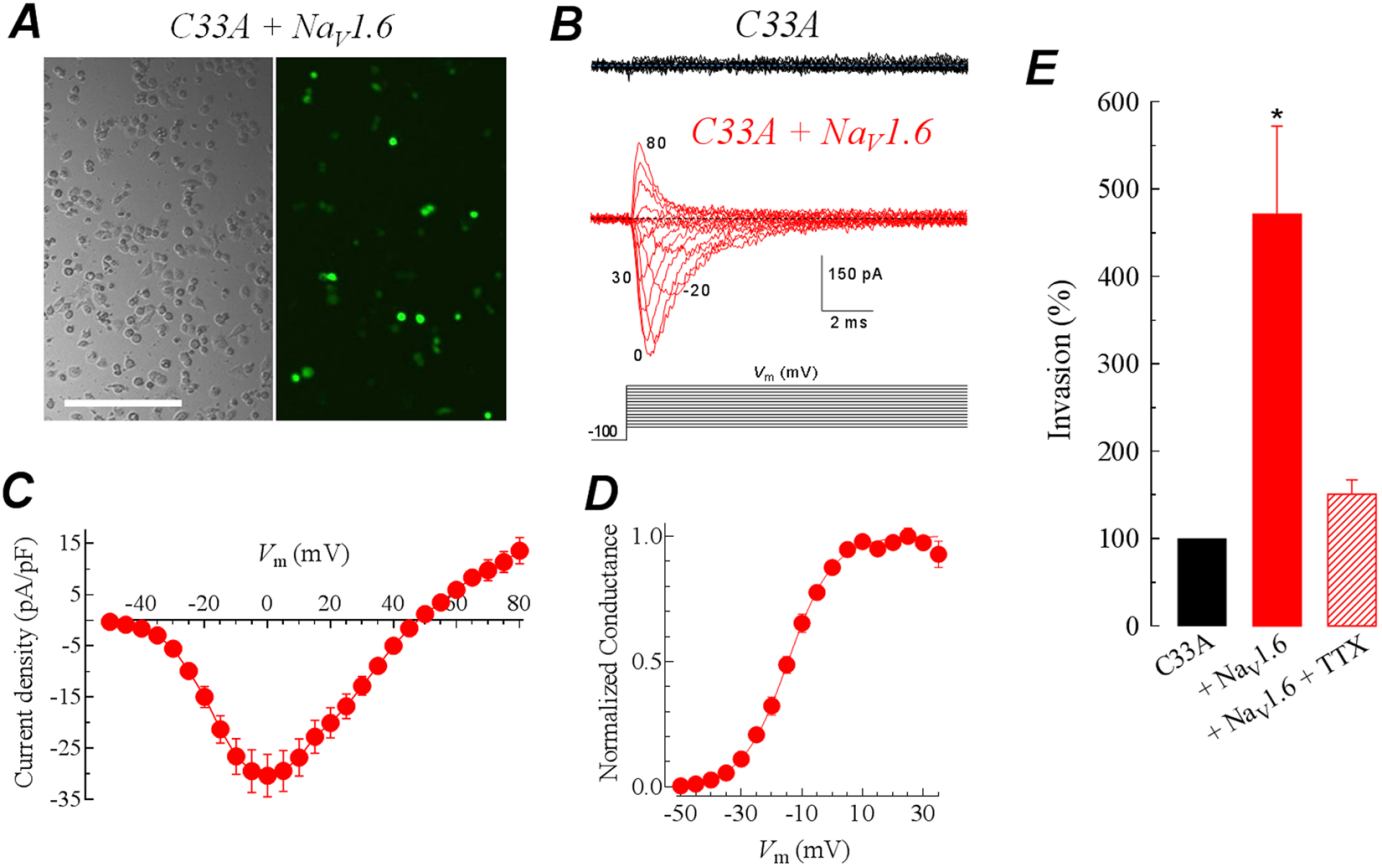
The heterologous expression of Na_V_1.6 channels boost the invasive capacity of cervical cancer cell lines. (**A**) Representative images of phase contrast and fluorescent microscopy of C33A cells 36 h after co-transfection with Na_V_1.6 and GFP cDNAs. GFP-fluorescence indicated that 50–60% cancer cells were positively transfected. (**B**) Representative families of sodium currents obtained from non-transfected (black traces) and transfected C33A cells (red traces) with the Na_V_1.6 channel in response to 16-ms pulses that depolarized the cell membrane from −80 to +80 mV in 10-mV steps applied every 10 s from a holding potential of −100 mV. Dotted lines indicate the baseline (zero current). Shown recordings are the average of two current traces at any given membrane potential and filtered at 5 kHz. (**C**) Current-voltage relationship for Na_V_1.6 channels heterologously expressed in C33A cells. Peak Na^+^ currents were averaged and plotted as a function of the depolarizing potential (*V*_m_). (**D**) Activation of normalized Na^+^ conductance. Same cells as in (**C**). Smooth line is the fit to a Boltzmann function (see Methods) with the following parameters: *V*_1/2_ = −12.2 ± 1.2 mV and *k* = 9.7 ± 1.0 mV; n = 9 cells. (**E**) The heterologous expression of Na_V_1.6 channels enhances the invasive capacity of C33A cells. Relative invasion of C33A cells transfected with Na_V_1.6 in absence or presence of 1 µM TTX, with respect to the control, untransfected C33A cells (black column). Columns represent the mean value of three independent experiments performed in triplicate (mean ± SD). *Statistically different from control condition (*P* < 0.05).

Because the absence of reliable voltage-gated sodium currents in CeCa cell lines, we performed transient transfections of cervical cancer cell lines with the Na_V_1.6 channel in order to explore its functional expression and role in the metastatic behavior of CeCa. Transfection efficiency was about 50–60% for C33A, and 30–35% for SiHa and HeLa cells (Fig. 5A). Transfected C33A cells generated very robust sodium currents in comparison with non-transfected cells (Fig. 5B). In addition, current-voltage relationship (Fig. 5C) and activation conductance (Fig. 5D) of heterologous Na_V_1.6 channels expressed in cervical cancer cells were similar to those previously reported from our group in primary cultures derived from CeCa biopsies^24^, and from other reports in neurons and HEK-293 cells^38,41^, including the high sensitivity to tetrodotoxin (TTX) of these channels (Supplementary Fig. S5). In order to evaluate the potential role of these transfected Na_V_1.6 channels in the metastatic behavior of the C33A cells, we performed *in vitro* invasion assays by using matrigel-invasion chambers. The cellular invasiveness of Na_V_1.6-transfected C33A cells was significantly increased (almost 5-fold) compared with control cells, this effect was prevented by the addition of 1 μM TTX to the culture media (Fig. 5E). These results support our previous observations regarding the contribution of Na_V_1.6 channels to the invasion capacity of CeCa primary cultures^25^, and that the heterologous expression of Na_V_1.6 channels is enough to promote TTX-sensitive invasion of a CeCa cell line positive to HPV16^42^. In addition, we also assessed cell proliferation by the MTT colorimetric assay and cell migration using the scratch-wound assay in the same cells transfected with the Na_V_1.6 plasmid; as expected, the results indicated that overexpression of Na_V_1.6 channels do not modify neither proliferation nor migration in CeCa cell lines (Supplementary Figs S7 and S8), indicating that Na_V_1.6 channel contribution on CeCa behavior is specific for cell invasiveness.

### The functional expression of Na_V_1.6 channels increases the specific secretion and activity of matrix metalloproteinase type 2

**Figure 6:**
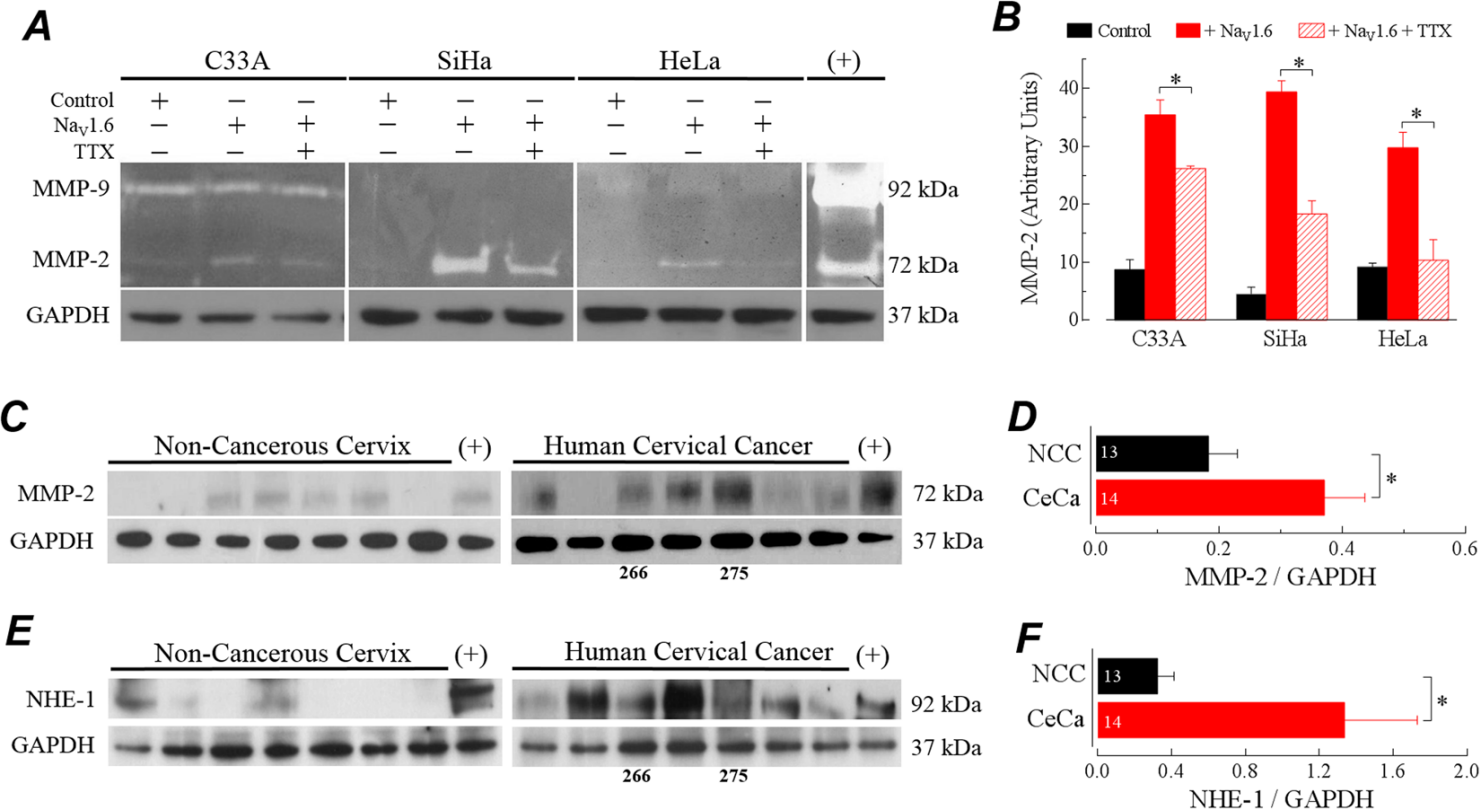
Na_V_1.6 channels activity induces secretion of MMP-2 in cervical cancer cell lines. (**A**) Gelatin zymography for conditioned medium from cervical cancer cell lines. C33A, SiHa and HeLa cells were transfected with Na_v_1.6 and grown for 24 h in absence or presence of 1 µM TTX. Conditioned medium was obtained and cells were lysed. Activity for gelatinases MMP-2 and MMP-9 was analyzed on equal volumes of concentrated conditioned medium by using gelatin-substrate polyacrylamide gel electrophoresis followed by an incubation in activity buffer and a staining with Coomassie blue. The conditioned medium obtained from MCF-7 cells treated with 100 ng/ml phorbol 12, 13-dibutyrate (PDB) for 40 h, was used as positive control. Proteolytic activity was detected as clear bands against a dark background of undigested substrate (*upper panel*). Total protein extracts from cell lysates were analyzed by western blotting with anti-GAPDH antibody (*bottom panel*). The results shown are representative of three independent experiments. (**B**) Secretion of MMP-2 was quantified by densitometry analysis using GAPDH bands for normalizing. Results are given as the amount of gelatin degradation showed as clear bands relative to GAPDH bands for each condition. Columns are means ± SD from three independent experiments. *Statistically significant as *P* < 0.05. (**C**) Representative western blot for MMP-2 expression in total protein extracts from human biopsies of NCC and CeCa. Blots were stripped and re-probed for total GAPDH as the loading control. (**D**) Expression of MMP-2 protein was studied by densitometry analysis of western blot experiments. Results are given as the amount of MMP-2 protein relative to that of GAPDH for NCC (*n* = 13) and CeCa (*n* = 14). Columns are means ± SEM. * Statistically significant as *P* < 0.05. (**E**) Western blot analysis of NHE-1 expression and (**F**) relative levels of NHE-1 protein in the same samples of panel (C) and (D), respectively. Data are means ± SEM, *P < 0.05. Samples used in (**C**) and (**E**) are exactly the same.

It is well known that matrix remodeling proteinases, such as matrix metalloproteinases (MMPs), play an important role in carcinoma cell invasion^6^. Therefore, we wonder whether heterologous expression of Na_V_1.6 channels in cervical cancer cells induces secretion of the gelatinases associated to cell invasion, *i.e*., MMP-2 and MMP-9. First, by using gelatin zymography and western blot experiments, we determined both basal proteolytic activity and protein expression of MMP2 and MMP9 in supernatants of C33A, SiHa and HeLa cell cultures, incubated in the presence of complete medium (DMEM + 10% FBS) or fasted conditioned medium (DMEM without FBS). We found robust proteolytic activity of MMP2 and MMP9 when CeCa cells were incubated in complete medium but there was no activity when cells were fasted during 24 or 48 h. In parallel with such observations, MMP2 and MMP9 proteins were only detected in the supernatants coming from complete medium condition of CeCa cell cultures (Supplementary Fig. S9). Then, we sought to examine the effect of overexpressing Na_V_1.6 channels in CeCa cell lines on proteolytic activity and expression of MMP2 and MMP9 when basal levels of these proteases are null, *i.e*., incubated under fasted conditioned medium at 48 h. Thus, C33A, SiHa and HeLa cells transfected with Na_V_1.6 channel were grown in conditioned medium for 48 h, after that, culture medium was recovered and cells were lysed. Secretion and activity of MMP-2 and MMP-9 were analyzed as a whole by gelatin zymography, whereas immunoblotting for GAPDH protein from cell lysates was used as a load control. In all three cervical cancer cells, regardless of whether or not they were positive to any type of HPV, we found degradation bands at 72 kDa, corresponding to the size of MMP-2, although with different intensities among the three CeCa cell lines (Fig. 6A). The activity of this metalloproteinase was more robust in SiHa cells (HPV-16), than in HeLa (HPV-18) or in C33A (negative to HPV). Regardless of the intensity, the activity of MMP-2 was significantly increased after cells were transfected with the Na_V_1.6 channel and the presence of 1 μM TTX in this last condition prevented partially this increase in MMP-activity (Fig. 6A,B). On the contrary, secretion and activity of MMP-9 was only observed in C33A cells without evident changes among treatments (Fig. 6A). These results suggest a potential role for the MMP-2 in the Na_V_1.6-mediated invasion of CeCa cell lines, therefore we look for the expression of this metalloproteinase in fresh cervical tissue samples. Western blot results showed that MMP-2 protein is expressed in total protein extracts from non-cancerous cervix and cervical cancer biopsies (Fig. 6C), however the immunoreactivity of the detected bands was around 2-fold more intense in CeCa samples (Fig. 6D). It has been shown that NHE-1, a regulator of intracellular pH, is essential for tumor invasive capacity^10,43,44^, and that its function play a role in the acidification needed for proteases activation^44–46^. Thus, we evaluated the expression of the NHE-1 in the same samples where we observed the up-regulation of the matrix metalloproteinase type 2. Western blots show the presence of NHE-1 protein in 4 out of 13 (31%) biopsies of NCC and in 9 out of 14 (64%) of CeCa biopsies (Fig. 6E). The densitometric analysis of protein bands suggest a 3-fold up-regulation in the relative expression of NHE-1 in CeCa biopsies compared with non-cancerous cervical tissue (Fig. 6F). Similar results were obtained for the evaluation of protein expression of the Na^+^/Ca^2+^ exchanger type 1(NCX-1; a likely modulator of the activity of NHE-1) in the same uterine cervix biopsies (Supplementary Fig. S10).

**Figure 7:**
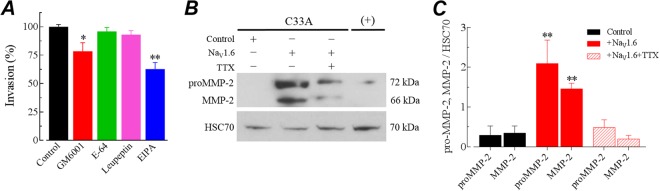
The promotion of CeCa cell invasiveness by Na_V_1.6 channels activity is mainly through secretion of pro- and mature MMP-2 forms. (**A**) Effect of protease inhibitors and EIPA on invasive capacity of Na_V_1.6-transfected C33A cells. Cells C33A transfected with Na_V_1.6 were seeded at cellular density of 6 × 10^4^ cells per insert in the absence (Control) or the presence of protease inhibitors (GM6001, 25 µM; E-64, 100 µM; Leupeptin, 100 µM), or the NHE-1 specific inhibitor (EIPA, 1 µM) using a serum gradient of 10% for 48 h. For these experiments, invasive cells were stained with DAPI, photographed and counted automatically. Results from six experimental observations of two independent experiments are expressed as relative invasion (mean ± SD), normalized to the control condition. Statistical difference at *P* < 0.05 for * and *P* < 0.01 for ** (Mann-Whitney Rank Sum test). (**B**) Representative western blotting experiment for the analysis of pro- and mature MMP-2 forms in supernatants of C33A cultures. Conditioned medium from C33A cells transfected with Na_V_1.6 and grown in the absence or the presence of 1 μM TTX was recovered after 48 h and concentrated by centrifugation. HSC70 was used as a loading control. Supernatants form C33A cells grown in complete medium (10% FBS) served as a positive control (+). (**C**) Evaluation of pro- and mature MMP-2 expression. Quantification was made by densitometric analysis of western blot images. Results are given as the amount of pro-MMP2 and MMP-2 protein relative to that of HSC70 in cell lysates. Columns are mean ± SD from three independent experiments. *Significantly different from Control at *P* < 0.01. There were no significant differences between proMMP-2 and MMP-2 forms in Na_V_1.6-transfected C33A cells (solid red columns).

Because several classes of proteases, additional to MMPs, have been involved in the degradation of the extracellular matrix (ECM) associated to cancer invasion and progression^9,47^, we assess whether the overexpression of Na_V_1.6 channel in cervical cancer cell lines could regulate the activity of such proteases. To address this issue, we performed invasion assays of Na_V_1.6-transfected C33A cells in the presence of different proteases inhibitors as GM6001 (broad spectrum MMP-inhibitor), cysteine cathepsins (E-64) and serine and threonine peptidases (Leupeptin). The results suggest that the invasiveness of C33A cells lead by the Na_V_1.6 activity could be specifically attributed to MMP activity, as only GM6001 significantly attenuates the increase in C33A cells invasion due to the overexpression of Na_V_1.6 channels in these cells (Fig. 7A). Interestingly, in accordance with this observation, a stronger attenuation in the amount of invasive cells was observed by using EIPA, an inhibitor of the NHE-1 exchanger, confirming the participation of the NHE-1 exchanger in the acidification of the extracellular matrix required for the activity of MMPs. Finally, in order to get some insights about the regulation of MMP-2 induced by the activity of Na_V_1.6 channels, we measured the levels of immature (proMMP-2) and mature (MMP-2) forms of this metalloproteinase in supernatants of Na_V_1.6-transfected C33A cell cultures in the absence and the presence of 1 µM TTX. The results are summarized in Fig. 7B,C. Western blotting analysis show that the overexpression of Na_V_1.6 channels in C33A cells induced robust increments in both the proMMP-2 (7-fold) and the mature form of MMP-2 (4-fold) in the supernatants, in comparison with plain C33A cells supernatants (Fig. 7C). These increments were prevented by the blocking of Na_V_1.6 channels with TTX.

## Discussion

During the last two decades new non-canonical, *i.e*. non-excitable, functions for voltage-gated sodium channels have emerged, mainly for the metastatic behavior of several epithelial carcinomas^9,47^. We previously show that Na_V_1.6 channel plays an important role for the invasiveness of cervical cancer primary cells. Even though several α-subunits of Na_V_ channels were expressed in biopsies and primary cultures of CeCa, only the messenger for Na_V_1.6 was over expressed (around 40-fold) in comparison with cervical non-cancerous cells^24,25^. The suppression of Na_V_1.6 channels activity resulted in a substantial reduction of cell invasion without affecting proliferation or migration parameters^25^. All these results were obtained by using biopsies positives to HPV-16, the most frequent viral type detected in CeCa, accounting for approximately 50% of all cases; however, at least eight more high-risk types of HPVs (18, 58, 33, 45, 31, 52 and 35) account for an additional 40% of CeCa cases^1,48^. In the present study we have found that the over expression of Na_V_1.6 channels is also conserved in CeCa biopsies positive to other high-risk HPV types, that together with our results in samples positive to HPV-16, allowed us to conclude that Na_V_1.6 channel over expression is a general characteristic of cervical carcinoma, regardless the associated HPV-type. In addition, the increase in RNA messenger was also accompanied with a significant up-regulation of the Na_V_1.6 channel protein in CeCa biopsies when compared with non-cancerous cervical tissue. Interestingly, the boost in the channel protein resulted in changes in the subcellular localization, as the strongest signal was observed in intracellular compartments in CeCa samples whereas in NCC tissue it was more abundant in the plasma membrane (Fig. 2). Thus, *SCN8A* gene is upregulated in most of human cervical cancer cases (around 90% of all) compared with both non-cancerous cervix and cervical intraepithelial neoplasia samples. Additionally, these deregulated *SCN8A* levels allowed to discriminate with high sensitivity and specificity cases of non-cancerous cervix from invasive cervical cancer (Supplementary Fig. S4). However, further studies are needed to determine the potential use of the expression levels of *SCN8A* as a predictive or prognostic molecular marker for human cervical cancer.

To further study the contribution of Na_V_1.6 channels to the invasiveness of cervical cancer^25^, we used commercially available CeCa cell lines; however, exhaustive patch-clamp whole-cell recordings in four different CeCa cell lines provided no evidence of voltage-gated sodium currents, except in C33A cells (characterized for being negative to any HPV type), which displays very small endogenous sodium currents (Fig. 5B). Despite this fact, our immunofluorescence, western blot and PCR experiments (Figs 3 and 4) provide strong evidences about the presence of Na_V_1.6 channels in intracellular compartments of CeCa cell lines, mainly in C33A, SiHa and HeLa cells. It has been shown previously in macrophages and melanoma cells that an isoform of Na_V_1.6 channel is expressed intracellularly, in vesicles that are distributed throughout the cytoplasm, but not at the plasma membrane^49^. The activity of these Na_V_1.6 channels was shown to regulate cellular invasion of macrophages and melanoma cells. The channel isoform was identified as a full-length splice variant of *SCN8A* that lacks exon 18 (Δ18)^23^. In agreement with these reports, our results show a stronger expression of the Δ18 variant in CeCa cell lines and biopsies than in NCC and CIN tissue samples (Fig. 4), suggesting a putative role of this variant in CeCa cellular invasion as shown for leukemia and melanoma cells^23^. This observation could be a partial explanation for the widely distributed immunoreactivity signal of Na_V_1.6 channel in cervical cancer cells and tissues compared with the localized plasma membrane signal in non-cancerous slice tissues (Figs 2 and 3), as the Δ18 variant of Na_V_1.6 channels has been localized only in intracellular compartments^23^. Furthermore, this could explain also the lack of plasma membrane currents in all CeCa cell lines that we explored in this work. In summary, the results from this section show for the first time the expression of *SCN8A* splice forms during progression of human cervical cancer. It is worth nothing that our previous results with CeCa primary cultures indicates a role for the Na_V_1.6 channels expressed in the plasma membrane, as blocking its activity with the Cn2 scorpion toxin decreased the invasiveness behavior of the CeCa primary culture cells lines^25^. Because Cn2 toxin is not membrane-permeable and its known mechanism of action consists in modifying Na_V_1.6 channels gating by binding to extracellular receptor sites^50,51^, the described effect on cell invasion must be due mainly to the interaction with the Na_V_1.6 channels locate at the plasma membrane of such CeCa tumor cells. In the case of the putative intracellular expression of Na_V_1.6 channels in CeCa cell lines, it remains to be confirmed if the channel is functional at the intracellular compartments and whether this has any contribution to the metastatic behavior of these cell lines. Also, these observations indicate that cell lines might not be fully representative of primary cultures derived from fresh tumor biopsies, as the latter showed plasma membrane voltage-activated sodium currents^25^, whereas in this study we demonstrated that, under our experimental conditions, CeCa cell lines lack these type of currents. A likely explanation for this discrepancy could be the experimental conditions, for instance, it has been shown that serum concentrations higher than 5% in the cell culture has a significant decrease in Na_V_s activity in rat prostate cancer cell line^52^; the CeCa cell lines we used here have been growing in 10% FBS conditions since they were immortalized. Therefore, it is likely that Na_V_ channels in plasma membrane of CeCa cell lines were down-regulated for serum and/or some other extracellular growth factors used in the daily propagation of these cell lines.

In this work we also initialized the study about the mechanism underlying the regulation of CeCa cell invasion mediated by Na_V_1.6 channels. We found that heterologously expressed Na_V_1.6 channels increase the invasive capacity cervical cancer cells. Furthermore, the inhibition of Na_V_1.6 channel activity by TTX, prevents the enhanced invasive capacity of CeCa cells (Fig. 5). Interestingly, the proliferation and migration properties of these cancer cells was not significantly affected (Supplementary Figs S7 and S8). These results are in agreement with previous data from our group and others suggesting that the activity of plasma membrane expressed Na_V_ channels is mainly associated to cell invasiveness in several types of cancer^10–15,17,25^, although a few reports indicate that Na_V_ channels also control cell proliferation and migration in astrocytoma, prostate and gastric cancer^13,53,54^. Recently, it has been shown that pro-invasive effect of Na_V_1.5 channel in breast cancer cells is modulated in part through enhancement of MMPs activity^55^. Matrix metalloproteinases (MMPs) are secreted proteases that induce degradation of various components of the basement membrane and extracellular matrix (ECM) including collagens, laminin, fibronectin, tenascin, elastin and proteoglycans^56^. It has been demonstrated that MMPs play a crucial role in tumor invasion and migration, as well as other cancer hallmarks^6,57^. Within the several MMPs associated to cancer invasion and progression, MMP-2 and MMP-9 have been particularly upregulated in several tumor types^58–60^. In the present work, we found that functional expression of Na_V_1.6 channels at the plasma membrane of cervical cancer cells enhances activity and protein expression of MMP-2 in a specific manner, as MMP-9 did not show changes in the proteolytic activity (Fig. 6). The activity of other proteases like cysteine cathepsins, and serine and threonine peptidases could be discarded also because specific inhibitors did not affected the invasion promoted by the overexpression of Na_V_1.6 channels in C33A cells (Fig. 7A). In addition, according to our western blot analysis of immature and mature forms of MMP-2, we propose that Na_V_1.6 activity in C33A cells promotes the secretion and the maturation (activity) of MMP-2 that leads the increase in invasiveness of this CeCa cells. In this regard, matrix metalloproteinase type 2 (MMP-2) and type 9 (MMP-9) have been found at the mature invadopodia of cancer cells where they are also secreted^61^, and also mediate colon cancer cell invasion^58^. Recent advances have highlighted the relevance of the extracellular acidification in the tumor microenvironment for cell invasive capacity and cancer cell propagation^10,44^. In this regard, the Na^+^/H^+^ exchanger-1 (NHE-1) is highly expressed in several tumors and has been implicated as necessary protein for cell invasion by regulating extracellular and intracellular pH^10,62,63^. In addition, a previous study reported that inhibition of Na_V_1.6 channels activity decreased the protein expression levels of NHE-1 in mouse microglial cells^64^. Therefore, these bibliographic data prompted us to question whether the enhanced activity and expression of MMP-2 in CeCa cells and tissue was accompanied by a change in the NHE-1 protein expression. Our western blots results confirmed that both proteins NHE-1, and also the Na^+^/Ca^2+^ exchanger (NCX-1), were upregulated in human cervical biopsies (Fig. 6E and Supplementary Fig. S10). Furthermore, the observation that invasion of Na_V_1.6-transfected C33A cells was significantly reduced by inhibit the function of NHE-1 (Fig. 7A), strongly suggests that acidification of the extracellular matrix is a crucial requirement for the activity of MMPs. Thereby highlighting a possible relationship between the Na_V_1.6 channels activity, the up regulation of the NHE-1 and the enhanced activity of MMP-2, that could lead to the observed increase in cell invasion of cervical cancer cells.

In conclusion, this study shows that *SCN8A* gene expression is upregulated in human cervical tissue only when cancer has been established. At this stage the alternative splicing of *SCN8A* gene favors the Δ18 variant over the neonatal and the adult variants. The invasive capacity of cervical cancer cells associated to Na_V_1.6 channels activity is mediated by a mechanism that involves the participation of NHE-1 and the specific secretion and proteolytic activity of MMP-2. The expression of Na_V_1.6 channels in cervical cancer could therefore represent a molecular target for reducing the metastasis of this carcinoma.

## Methods

### Ethics Statement

This research protocol was approved by the Scientific and Ethics Committees of the Hospital General de México (approval number DIC/03/311/04/051) and was performed in accordance with the ethical principles described in the 1964 Declaration of Helsinki. Informed written consent was obtained from all participants prior to their inclusion in the study.

### Human Biopsies

This study included 57 samples from cervical cancer patients distributed as follows: 35 positive to HPV16; 22 positive for the other major HPV oncogenic types (HPV18, 31, 45, 52, 58, 59 and 68); 26 samples from low-grade cervical intraepithelial neoplasia (CIN-1); 17 from high-grade cervical intraepithelial neoplasia (CIN-2/3); and 19 non-cancerous cervical samples (NCC). All samples were obtained at the Oncology Unit of the Hospital General de Mexico, and they were selected from patients with an incident case and who had not received any anticancer therapy. Non-cancerous cervical biopsies were obtained by hysterectomy from patients with uterine myomatosis and with Pap smear test negative to cancer. HPV detection and typing in all specimens were performed as described previously^65^. All patients were histopathologically diagnosed and the cervical cancer stage was determined according to the International Federation of Gynecology and Obstetrics (FIGO) staging system. After sampling, all patients were referred for specific treatments according to the guidelines of the American Cancer Society.

### RNA extraction, Reverse Transcription and Quantitative Real Time PCR (qRT-PCR)

Biopsies from human cervical tissue were surgically sampling using a Schubert tweezer. Each biopsy was placed immediately in a sterile plastic Petri dish containing 4 °C Hank’s solution supplemented with 2 mM glutamine, penicillin (50 U/ml) and streptomycin (50 µg/ml). The biopsies were transported on ice to the laboratory for processing. Samples were transferred into a sterile Petri dish to remove and discard damaged areas using a scalpel. Each tissue sample was cut in small pieces of approximately 5-mm and then transferred into a mortar containing liquid nitrogen followed by a vigorous trituration until get a fine powder of tissue. Finally, an adequate volume of TRIzol Reagent (Thermo Fisher Scientific; Waltham, MA) was added to the pulverized tissue and total RNA isolation was performed according to the manufacturer’s protocol. Total RNA from cell lines was obtained using the same procedure. After total RNA isolation the remaining interphase and the organic phenol-chloroform phase were stored for further DNA and protein isolation. The RNA integrity was confirmed by agarose gel electrophoresis by the presence of 28S and 18S ribosomal bands; while RNA yield and purity were determined by spectrophotometry and only those samples with an A_260_/A_280_ ratio above 1.6 were kept for further experiments. Reverse transcription for 3 µg of total RNA was carried out using the High-Capacity kit (Applied Biosystems; Foster City, CA) in 20 µl of final volume according to the manufacturer’s specifications. Gene expression of *SCN8A* was assessed in all samples by quantitative RT-PCR using TaqMan probes (Applied Biosystems), as we have reported previously^25^. Genes for 18S, GAPDH and HPRT1 were used as internal controls. Experiments were run in triplicates in a final volume of 20 µl, including 200 ng of cDNA template, 10 µl of 2× TaqMan Universal PCR Master Mix (Applied Biosystems), 1 µl of 20× TaqMan Gene Expression Assay and 7 µl of water. Cycling was carried out using a Rotor-Gene thermocycler (Qiagen; Hilden, Germany) with the following conditions: a PCR activation step at 50 °C for 2 min followed by 95 °C for 10 min, then 40 cycles of melting at 95 °C for 15 s and annealing/extension at 60 °C for 1 min. Evaluation of gene expression was based on relative standard curves constructed from a 10-fold serially diluted pool of CeCa cDNAs ranging from 500 to 0.05 ng. The expression of *SCN8A* gene was normalized in each sample to the expression of internal standard gene and relative expression levels were calculated using comparative 2^−ΔΔ*CT*^ method as described previously^25^.

### Identification of *SCN8A* splice variants in human cervical tissue

Total RNA from human biopsies of non-cancerous cervix, cervical intraepithelial neoplasia, invasive cervical cancer and cervical cancer cell lines was isolated using the TRIzol Reagent. First strand cDNA synthesis was performed from 3 µg total RNA using the High-Capacity kit (Applied Biosystems) in 20 µl of final volume according to the manufacturer’s specifications. The first strand cDNA product (250 ng) was used as template in 10 µl PCR reactions with final concentrations of 200 µM dNTP, 0.3 µM primers for *SCN8A* Exon 18 (forward primer 5′-AAGTGGACAGCCTATGGCTTCG-3′, reverse primer 5′-TGTTGACATCTTCAATTTCAAATCGG-3′), 1.5 mM MgCl_2_, and 1.3 U of enzyme mix (Expand High Fidelity PCR System, Roche Diagnostics; Mannheim, Germany). A single round of amplification (35 cycles) was initiated by denaturation for 2 min at 95 °C followed by 45 sec at 95 °C, 30 sec at 60 °C and 30 sec at 72 °C. A final extension step by 4 min at 72 °C was included. PCR products of *SCN8A* splice variants were electrophoretically separated in 2% agarose gels and visualized by ethidium bromide fluorescence. The identity of each splice variant was confirmed by automated sequencing. Full scan of agarose gels are shown in Supplementary Fig. S11.

### Culture of cell lines

Human cervical cancer cell lines purchased from the ATCC catalogue were used for studying mechanistic aspects. HeLa (positive to HPV18), SiHa and CaSki (positive to HPV16) and C33A cells (negative to any HPV) were used as cervical cancer models while ordinary HEK-293 and HEK-293 stably expressing human Na_V_1.6 channels were used as non-cancerous models. All cells lines were maintained in Dulbecco’s Modified Eagle Medium (DMEM) supplemented with 10% heat inactivated fetal bovine serum (FBS), 2 mM L-glutamine and penicillin-streptomycin (Gibco-Thermo Fisher Scientific) at 37 °C in a CO_2_ incubator.

### Protein Extraction and Western Blotting Experiments

Total protein extracts from human cervical tissues were obtained from stored phases after RNA isolation according to the Protein Isolation Procedure of TRIzol Reagent´s user guide. On the other hand, to prepare protein extracts from cell lines, cells were rinsed twice with phosphate-buffered saline (PBS) and lysed in the presence of RIPA buffer (25 mM Tris-HCl, pH 7; 150 mM NaCl, 1% IGEPAL, 1% sodium deoxycholate, and 1% sodium dodecyl sulfate), containing protease inhibitors cocktail (Roche Diagnostics). Cell lysates were sonicated in a Digital Sonifier (Branson Ultrasonics Corp.; Danbury, CT), with 4 short bursts (at 10% amplitude) of 10 sec followed by intervals of 60 sec for cooling on ice. Protein integrity was verified by SDS-PAGE followed by Coomassie’s blue staining. Total protein was quantified by Bradford Protein Assay (Bio-Rad; Hercules, CA) using ultra-pure bovine serum albumin (BSA) as standard. For western blotting experiments a 20-µg samples of protein homogenates were heated at 75 °C for 3 min, and then protein samples were separated by 8% SDS-PAGE under reducing conditions and then transferred to polyvinylidene fluoride membranes (Millipore; Burlington, MA). The membranes were blocked with 5% non-fat milk in TBST buffer (100 mM Tris, 150 mM NaCl and 0.1% Tween-20) for 3 hours. Work dilutions for primary antibodies were as follow: anti-Na_V_1.6, 1:5000 (Alomone Labs, Israel; ASC-009); anti-GAPDH, 1:1000 (GeneTex; Irvine, CA; GTX100118); anti-HSC70, 1:1000 (Santa Cruz Biotechnology, SC-7298); anti-Na^+^/Ca^2+^ exchanger (NCX), 1:1000 (GeneTex, GTX80928); anti-Histone-3, 1:1000 (GeneTex, GTX122148); anti-MMP2, 1:1000 (GeneTex, GTX104577); anti-MMP9, 1:1000 (GeneTex, GTX100458); anti-proMMP2, 1:1000 (Abcam, AB37150) and anti-NHE-1, 1:800 (GeneTex, GTX85046). Incubation with primary antibodies was performed overnight at 4 °C on a shaker. Membranes were then washed three times for 15-min continuous stirring each with TBST and incubated with secondary antibodies (1:10000) for 1 hour at room temperature. After washing again the blots with TBST, immunodetection was performed using electrochemiluminescense-plus kit (Thermo Fisher Scientific), and protein bands signal was captured on Kodak Bio-Mark MS films. Finally, X-ray films with protein bands were digitalized by a scanner and stored as high-quality images. Densitometry analysis of protein bands was performed using the Gel Tool from Fiji software^66^. Full scan of western blots are shown in Supplementary Fig. S11.

### Preparation of cytoplasmic and nuclear extracts

Cervical cancer cells were grown in 100-mm Petri dish until cells reached 90% confluence. Cells were then lysed with 200-µl *cytoplasmic buffer* (10 mM Tris-HCl, pH 7.4; 10 mM NaCl, 6 mM MgCl_2_, 10 mM NaF, 1 mM Na_3_VO_4_, 1 mM DTT, and 1 mM PMSF), and stirred for 5 min at 4 °C and then pelleted at 2600 rpm for 15 min at 4 °C. Supernatants recovered were labeled as cytoplasmic fraction. Pellets were washed twice with ice cold PBS. Pellets were resuspended in 40-µl *nuclear buffer* (20 mM HEPES, pH 7.9; 420 mM NaCl, 20% glycerol, 1.5 mM MgCl_2_, 0.2 mM EDTA, 1 mM Na_3_VO_4_, 10 mM NaF, 1 mM DTT, 0.2 mM PMSF), and vortexed for 15 min at 4 °C. Nuclear extracts were recovered by centrifugation at 12,000 rpm for 15 min at 4 °C.

### Immunohistochemistry

The protein expression of the Na_V_1.6 channels in the human cervical tissues was analyzed by standard immunohistochemistry procedure. Several homemade tissue microarrays (TMA) were built containing samples from dysplasia, tumor and non-cancerous tissue at the same slide. TMA stained by H&E were analyzed in blind conditions by two pathologist experts for proper staging. After deparaffinization and rehydration of paraffin-embedded tissue, the sections were treated with a high-pH (Tris buffer/EDTA, pH 9.0) target retrieval procedure (Dako PT-link, Dako; Santa Clara, CA). Endogenous peroxidase was then blocked by a commercial solution (Dako REAL, Dako) and incubated overnight at 4 °C with a rabbit-anti-Na_V_1.6 primary antibody (1:150; Alomone). Sections were then incubated with HRP-conjugated anti-rabbit secondary antibody (GeneTex) for 1 hour at room temperature. Immunoreaction was finally reveled by oxidation of 3–3’diaminobenzidine solution (Dako) during 5 min. Positive reaction was identified by a dark-brown precipitate. To determine the degree of protein expression in tissues, a qualitative scale was used, for negative (−), weak (+) and strong (++) signal intensity. A unique score was given per core. Negative controls were obtained using an irrelevant antibody instead of anti-Na_V_1.6 primary antibody. Finally, the slides were counterstained with hematoxylin and subjected to a fast dehydration process. Digital images were acquired using a light-transmitted microscope Leica ICC50 (Leica Microsystems; Wetzlar, Germany) and stored as high-quality images for documentation and later analysis.

### Immunofluorescence and Confocal Microscopy

For cell monolayer immunofluorescence experiments, five thousand cells were seeded on coverslips or 35 mm glass bottom dish (Ibidi; Munich, Germany) and incubated at 37 °C, 5% CO_2_ for 24 h. Cell monolayers were rinsed twice with PBS, fixed with 3.7% ice-cold paraformaldehyde in PBS. Tissue slides and cell monolayers were permeabilized with PBS supplemented with 100 mM glycine, 1% BSA and 0.2% Triton X-100 at room temperature for 30 min in a shaker. After three washes with PBS for 5 min each, preparations were blocked for 1 hour with PBS supplemented with 300 mM glycine, 3% BSA and 0.2% Triton X-100 at room temperature under continuous stirring. Primary antibody was diluted in PBS supplemented with 10% FBS and 0.2% Triton X-100 and incubated overnight at 4 °C in a humidified chamber. Three washes with PBS for 10 min each were performed for removing non-specific primary antibody binding. FITC-coupled secondary antibody was diluted in PBS supplemented with 10% FBS and 0.2% Triton X-100 and incubated for 1 hour at room temperature. Again, preparations were washed three times with PBS, incubated with DAPI for 5 min, and washed again with PBS before sealed with Dako Fluorescent Mounting Medium (Dako), while preparations from 35 mm glass bottom dish were maintained in PBS. Negative controls were obtained by pre-incubation of anti-Na_V_1.6 with the immunogenic peptide before primary antibody incubation step. Preparations were observed using a Zeiss LSM 800 confocal microscope with 405 nm and 514 nm laser lines, imaged through a 63× DIC 1.4 oil objective. Images were acquired with sequential excitation as stacks with 0.3 μm z-spacing. Detector gain and laser power were kept constant for all samples.

### Electrophysiology

Cervical cancer cells were co-transfected with plasmids containing Na_V_1.6 and GFP genes using JetPEI reagent (Polyplus transfection^TM^; Illrich, France). After transfection, cells were cultured for 36 h before electrophysiological experiments. Transfected cells were trypsinized and seeded on coverslips contained into a 35-mm Petri dish. Electrophysiological recordings were carried out between 2 and 10 h after seeding. The macroscopic activity of Na_V_1.6 channels was examined using the whole-cell configuration of the patch-clamp technique. Sodium currents were obtained at 21 °C using an Axopatch 200B amplifier, a Digidata1322a A/D converter and pCLAMP 9.4 software (Molecular Devices; Sunnyvale, CA). Currents were digitalized at 10 to 20 kHz, after 5 kHz analogue filtering. Whole-cell series resistance and cell capacitance were estimated from optimal cancellation of the capacitive transients with the built-in circuitry of the amplifier and in some cases was compensated electrically by 60 to 70%. Cells were bathed in a solution containing the following composition (in mM): 158 NaCl, 2 CaCl_2_, 2 MgCl_2_ and 10 HEPES-NaOH (pH 7.4). Internal recording solution contained the following composition (in mM): 106 CsCl, 30 NaCl, 1 CaCl_2_, 1 MgCl_2_, 10 EGTA and 10 HEPES-CsOH (pH 7.3).

Voltage-gated sodium currents were evoked by 16-ms depolarizing pulses to 0 mV from a holding potential of −100 mV applied every 10 s. The protocol used to build sodium current-voltage (*I-V*) relationships was as follows: plasma membrane was held at −100 mV and then stepped to potentials from −80 to +80 mV, in 5-mV increments, by applying brief (16 ms) depolarizations at a frequency of 0.1 Hz. Current amplitudes were normalized to cell capacitance and expressed as current density (pA/pF). Activation curves of Na^+^ channels were constructed by calculating the Na^+^ conductance at each test potential by dividing peak current amplitude by the respective driving force (*V*_m_ − *V*_rev_). Then, conductance was normalized to its maximal value and plotted against *V*_m_. Data points were fitted with a Boltzmann function: *G* = *G*_max_/(1 + exp (−(*V*_m_ − *V*_1/2_)/k)), where *G*_max_ is the maximum Na^+^ conductance; *V*_m_ is the test potential, *V*_1/2_ is the mid-point of activation, and *k* is the slope factor.

### Invasion Assay

Invasion assays were performed using the Corning^®^ Matrigel^®^ 24-well collagen-based cell invasion chamber with 8-µm pore size as described previously^25^. Briefly, cervical cancer cells were co-transfected with plasmids containing Na_V_1.6 and GFP genes and seeded in the inserts at 1 × 10^5^ cells density using culture medium with 5% FBS in absence or presence of 1-µM TTX, or in presence of protease inhibitors: GM6001, 25 µM (Millipore); E-64, 100 µM (Calbiochem; San Diego, CA); Leupeptin, 100 µM (Thermo Fisher Scientific), or the NHE-1 specific inhibitor 5-(*N*-ethyl-*N*-isopropyl) amiloride, EIPA, 1 µM (Sigma-Aldrich; St. Louis, MO). The lower chamber contained 500 µl of enriched culture medium with 15% FBS. Cancer cells transfected only with GFP and undergone trough the same procedure were used as a control. Chambers were incubated for 48 h at 37 °C in a 5% CO_2_ atmosphere. Cells on upper surface of membranes were removed with cotton swabs. Cells on the lower surface were incubated for 3 h in 400 µl fresh culture medium supplemented with MTT reagent. After incubation, culture medium was removed and tetrazolium salts formed were dissolved with 350 µl DMSO. Number of invasive cells was estimated by absorbance measurement at 570 nm, or in a few cases, invasive cells were fixed with absolute methanol at −20 °C for 10 min, stained with DAPI and photographed using an inverted microscope. Cells were counted automatically using the bright-clustered spot detection tool from NIS-Elements Advanced Research Imaging Software (Nikon, Japan). In all cases, three independent experiments were performed in triplicates.

### Scratch-Wound Assay

Monolayer cultures of cervical cancer cells co-transfected with plasmids containing Na_V_1.6 and GFP genes were treated for 2 h with 12 µM mitomycin C, then they were scratch-wounded using a sterile 10 µl pipette tip, washed twice with PBS and fed with fresh culture medium in the absence or the presence of 1 µM TTX. Cancer cells transfected only with GFP and undergone to the same procedure were used as a control. Simultaneously, cervical cancer cells without undergoing to transfection procedure and incubated in high-serum culture medium were used as positive control. Three independent experiments were performed in triplicates. Progress of cell migration into the wound was photographed using an IX71 Olympus inverted microscope coupled to an acquisition system Evolution VF Fast Cooled Color Camera. Cell migration (expressed as the migration rate: original scratch area - new scratch area)/original scratch área ×100%) was quantified using the polygon tool from Fiji software^66^.

### Proliferation Assay

Cervical cancer cells co-transfected with plasmids containing Na_V_1.6 and GFP genes were seeded by triplicate at 5 × 10^3^ cells/well in a 48-well plate and grown for a total of 96 h in absence or presence of 1 µM TTX. Cancer cells transfected only with GFP and undergone to the same procedure were used as a control. Culture medium and TTX were changed every day. Cell proliferation was measured by the tetrazolium salt assay. Briefly, MTT reagent was added after each incubation time and incubated for 3 h. Then, culture medium was removed and tetrazolium salts were dissolved with 300 µl DMSO. Cell proliferation was estimated by absorbance measurement at 570 nm using DMSO as blank. Three independent experiments were performed.

### Zymography

Conditioned mediums obtained from cervical cancer cells co-transfected with plasmids containing Na_V_1.6 and GFP genes were concentrated using Amicon^®^ ultra centrifuge filters (Merck Millipore). A conditioned medium as positive control was obtained from MCF-7 cells treated with 100 ng/ml phorbol 12, 13-dibutyrate (PDB) for 40 h. Equal volume of non-heated samples were mixed with sample buffer (2.5% SDS, 1% sucrose, 4-µg/ml phenol red) without reducing agents. Samples were electrophoretically separated in 8% polyacrylamide gels copolymerized with gelatin (1 mg/ml). Gels were rinsed twice with 2.5% Triton X-100 and incubated in activity buffer (50 mM Tris-HCl, pH 7.4; and 5 mM CaCl_2_) at 37 °C for 48 h. Gels were fixed and stained with 0.25% Coomassie Brilliant Blue G-250 in 10% acetic acid and 30% methanol. Proteolytic activity was detected as clear bands against the background blue stain of undigested substrate. Full scan of gelatin zymographies are shown in Supplementary Fig. S11.

### Statistical analysis

Quantitative results are given as the mean ± standard error (SEM) or standard deviation (SD) Differences in means were tested with an unpaired two-tailed Student’s *t* test and were accepted as significant if *P* < 0.05. Alternatively, a Mann-Whitney U test was used when the variance homogeneity test failed. Receiver Operator Characteristic (ROC) curve analysis was performed to select the best cut-off points to distinguish invasive tumors from controls as described previously^65^. Data analysis and graphing were performed using GraphPad Prism version 6.01 for Windows (La Jolla, CA) and SPSS version 17.0 software.

## Electronic supplementary material

Supplementary Information

## Data Availability

The datasets generated during and/or analysed during the current study are available from the corresponding author upon reasonable request.
