# Supplementary material for: The invasiveness of human cervical cancer associated to the function of NaV1.6 channels is mediated by MMP-2 activity
Source: Sci Rep. 2018 Aug 29;8:12995. doi: 10.1038/s41598-018-31364-y (PMC6115389; doi:10.1038/s41598-018-31364-y)

The invasiveness of human cervical cancer associated to the function of $Na_V1.6$ channels is mediated by MMP-2 activity

Osbaldo Lopez-Charcas[1], Ana Maria Espinosa[2], Ana Alfaro[2], Zazil Herrera-Carrillo[1], Belen Ernestina Ramirez-Cordero[1], Pedro Cortes-Reynosa[3], Eduardo Perez Salazar[3], Jaime Berumen[2], and Juan Carlos Gomora[1,*]

[1]Departamento de Neuropatología Molecular, Instituto de Fisiología Celular, Universidad Nacional Autónoma de México, Ciudad de México, 04510, México
Osbaldo Lopez-Charcas, Zazil Herrera-Carrillo, Belen Ernestina Ramirez-Cordero & Juan Carlos Gomora

[2]Unidad de Medicina Genómica, Facultad de Medicina, Universidad Nacional Autónoma de México/Hospital General de México, Ciudad de México, 06720, México.
Ana Maria Espinosa, Ana Alfaro & Jaime Berumen

[3]Departamento de Biología Celular, Cinvestav-IPN, Av. IPN # 2508, San Pedro Zacatenco, Ciudad de México, 07360, México.
Pedro Cortes-Reynosa & Eduardo Perez Salazar

*Correspondence to: Juan Carlos Gomora, Departamento de Neuropatología Molecular, Instituto de Fisiología Celular, UNAM, Mexico City, 04510, México, Tel. + (52-55)-5622-5752, Fax: + (52-55)-5622-5607, E-mail: jgomora@ifc.unam.mx

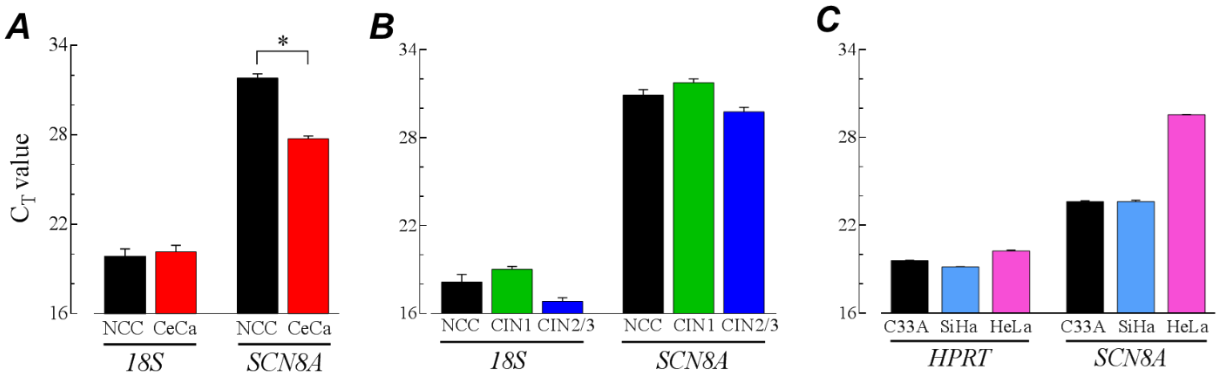

Supplementary Figure 1. Expression levels for *18S, HPRT* and *SCN8A* genes in biopsies from human cervical tissue and cervical cancer cell lines. (**A**) Average Ct values (mean ± SEM) for *18S* and *SCN8A* genes expressed in non-cancerous cervix, NCC (n = 20) *versus* invasive cervical cancer CeCa (n = 57). (**B**) Average Ct values for *18S* and *SCN8A* genes expressed in non-cancerous cervix *versus* low- and high-grade cervical neoplasia (CIN1 n = 23, CIN2/3 n = 16). *$P < 0.05$. (**C**) Average Ct values for *HPRT* and *SCN8A* genes expressed in the indicated cervical cancer cell lines (n = 3).

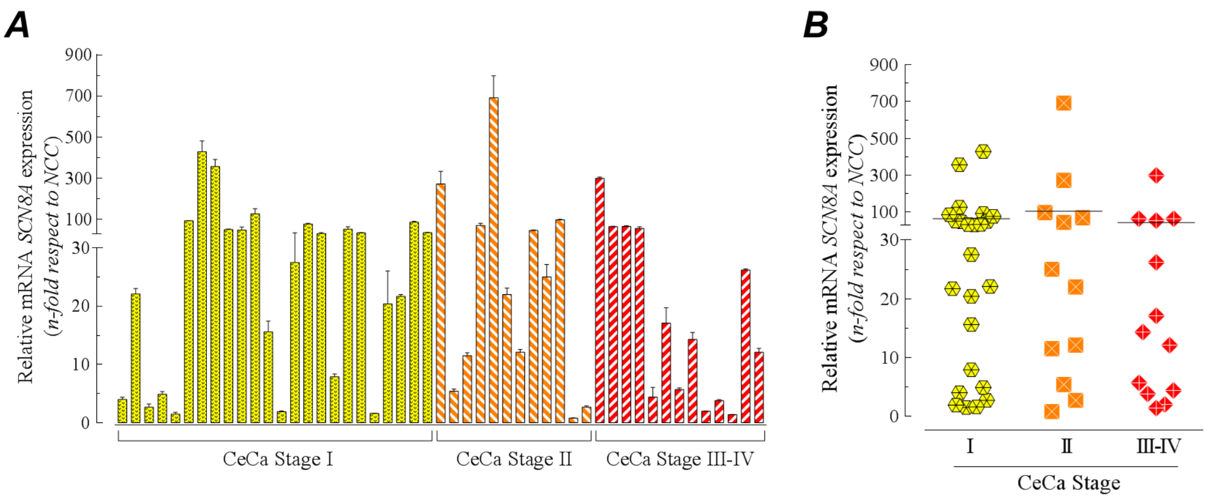

Supplementary Figure 2. Expression levels of *SCN8A* gene in cervical cancer biopsies staged according to the FIGO system. (**A**) Human CeCa samples positive to the main oncogenic HPV types (HPV16, 18, 31, 45, 52, 58, 59 and 68) were studied by quantitative PCR. Values are averaged fold-change ratio ($2^{-\Delta\Delta Ct}$) of *SCN8A* gene for each CeCa sample grouped according to the FIGO system. (**B**) Scattering of fold-change values for each group of samples. Horizontal black line represent the fold-change mean value. Expression of *SCN8A* gene was not significantly different between FIGO stages.

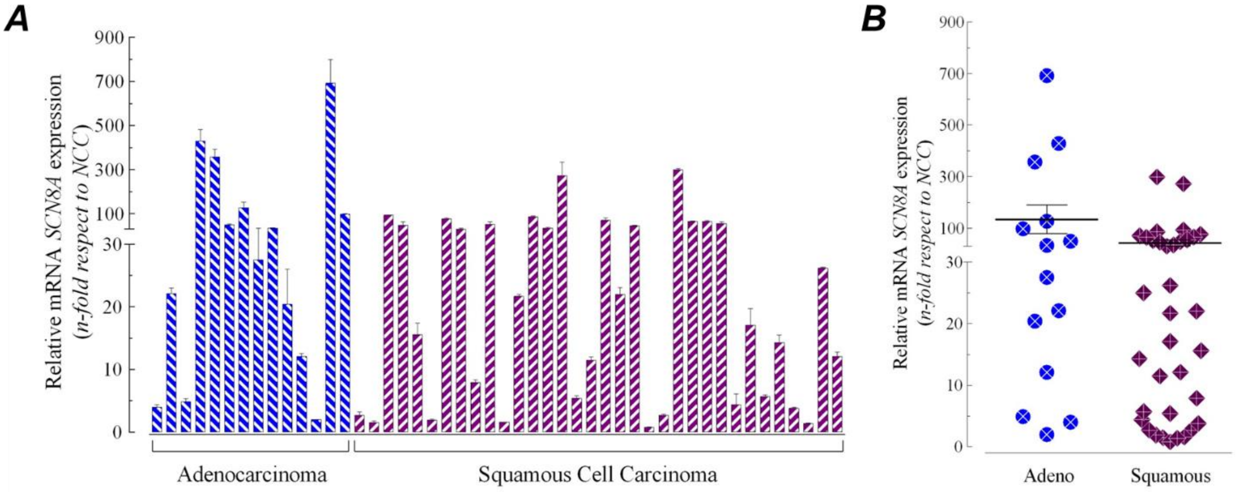

Supplementary Figure 3. Expression levels of *SCN8A* gene and histological type of cervical cancer. (**A**) Values of $2^{-\Delta\Delta Ct}$ from samples of CeCa positive to the main oncogenic HPV types (16, 18, 31, 45, 52, 58, 59 and 68) plotted as a function of the histological classification: adenocarcinoma or squamous cells carcinoma. (**B**) Scattering of fold-change values for each group of samples. Horizontal black line represent the fold-change mean value. Expression levels of *SCN8A* gene was not significantly different between adenocarcinoma and squamous cell carcinoma ($P < 0.05$).

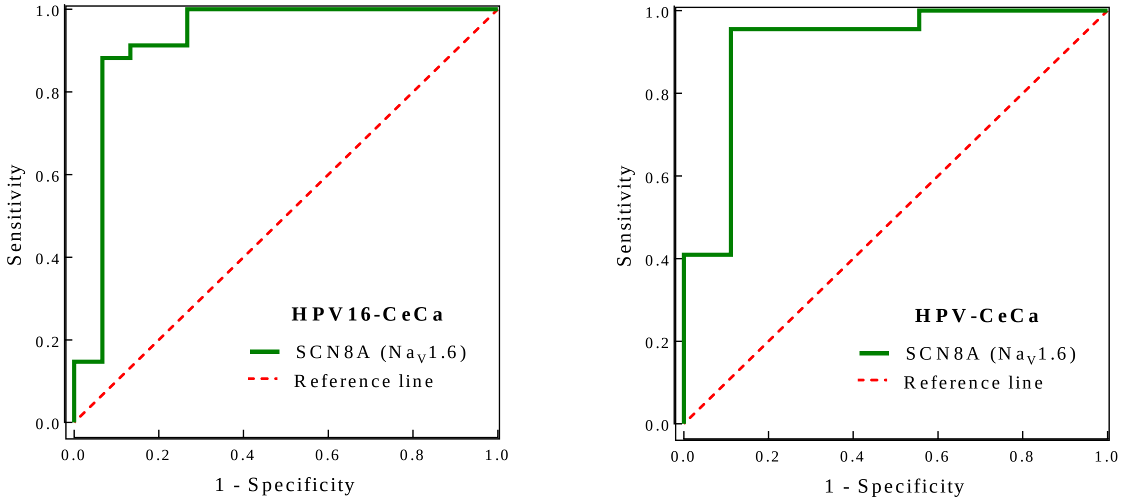

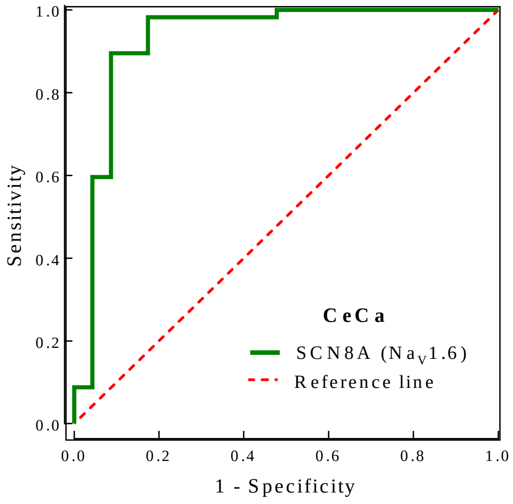

	AUC	SEM	Specificity (%)	Sensitivity (%)
HPV16-CeCa	0.924	0.056	92.4	85.7
HPV-CeCa	0.914	0.066	88.9	95.5
CeCa	0.924	0.042	82.6	98.2

Supplementary Figure 4. Receiver Operating Characteristic (ROC) analysis for *SCN8A* gene expressed in human cervical tissue. The expression levels of *SCN8A* gene in non-cancerous cervix, NCC (n = 20), were correlated to those obtained from cervical cancer positive to HPV16, (HPV16-CeCa, n = 35) and cervical cancer positive to another oncogenic HPV types, (HPV-CeCa, n = 22) in order to obtain the ROC curves. Shown are ROC curves for cervical cancer positive to HPV16 (**A**), cervical cancer positive to another oncogenic HPV types (**B**) and when all CeCa cases were grouped together (**C**). The inset table summarizes ROC parameters such as area under curve (AUC), specificity and sensitivity for each analysis.

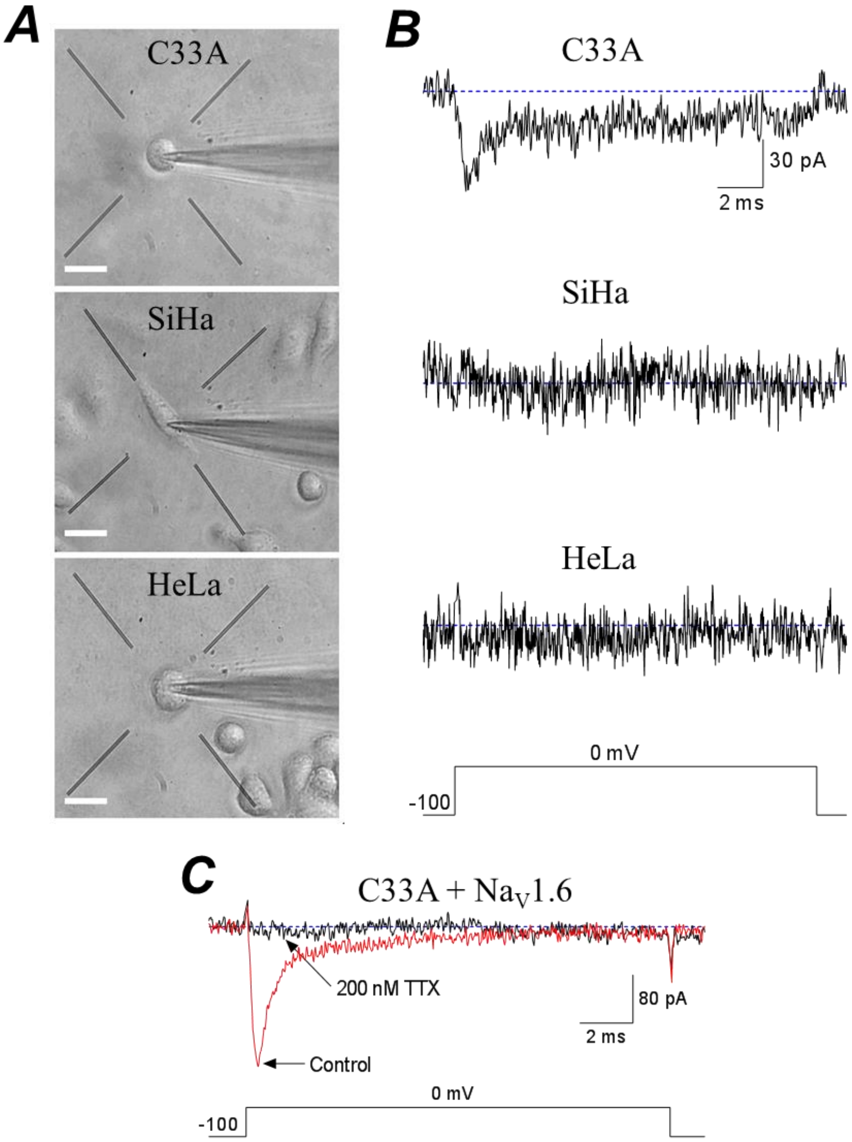

Supplementary Figure 5. Human cervical cancer cells do not express or express a tiny voltage-activated sodium current. (**A**) Microphotographs from C33A, SiHa and HeLa cells during electrophysiological recordings with the patch-clamp technique. (**B**) Expression of a tiny voltage-activated inward component in C33A cells (−4.1 pA/pF ± 0.8; n = 5), and the absence of this in SiHa and HeLa cells in response to depolarizing pulses at 0 mV from a holding potential of −100 mV. The percentage of C33A cells with current was rather low, 11% (5 out of 47 cells investigated). (**C**) TTX-sensitivity nature of $Na_V1.6$ channels heterologously expressed in C33A cells. Representative whole-cell voltage-gated sodium currents recorded at 0 mV in absence (*Control*) and presence of TTX (*200 nM TTX*). The completely block of the sodium current with this concentration of TTX agrees with the TTX-sensitivity of the $Na_V1.6$ channels.

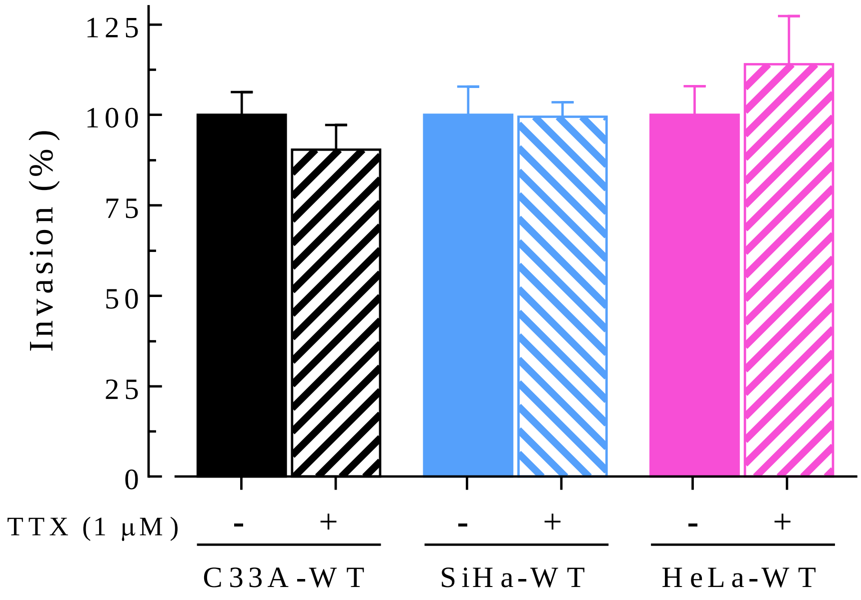

Supplementary Figure 6. Basal invasiveness of human cervical cancer cells. C33A, SiHa and HeLa cells were seeded at cellular density of 6×10^4 cells per insert in absence or presence of 1 µM TTX using a serum gradient of 10% for 48 h. Columns represent the mean value and are expressed as relative invasion (mean ± SD; *n* = 3), normalized to the Control condition.

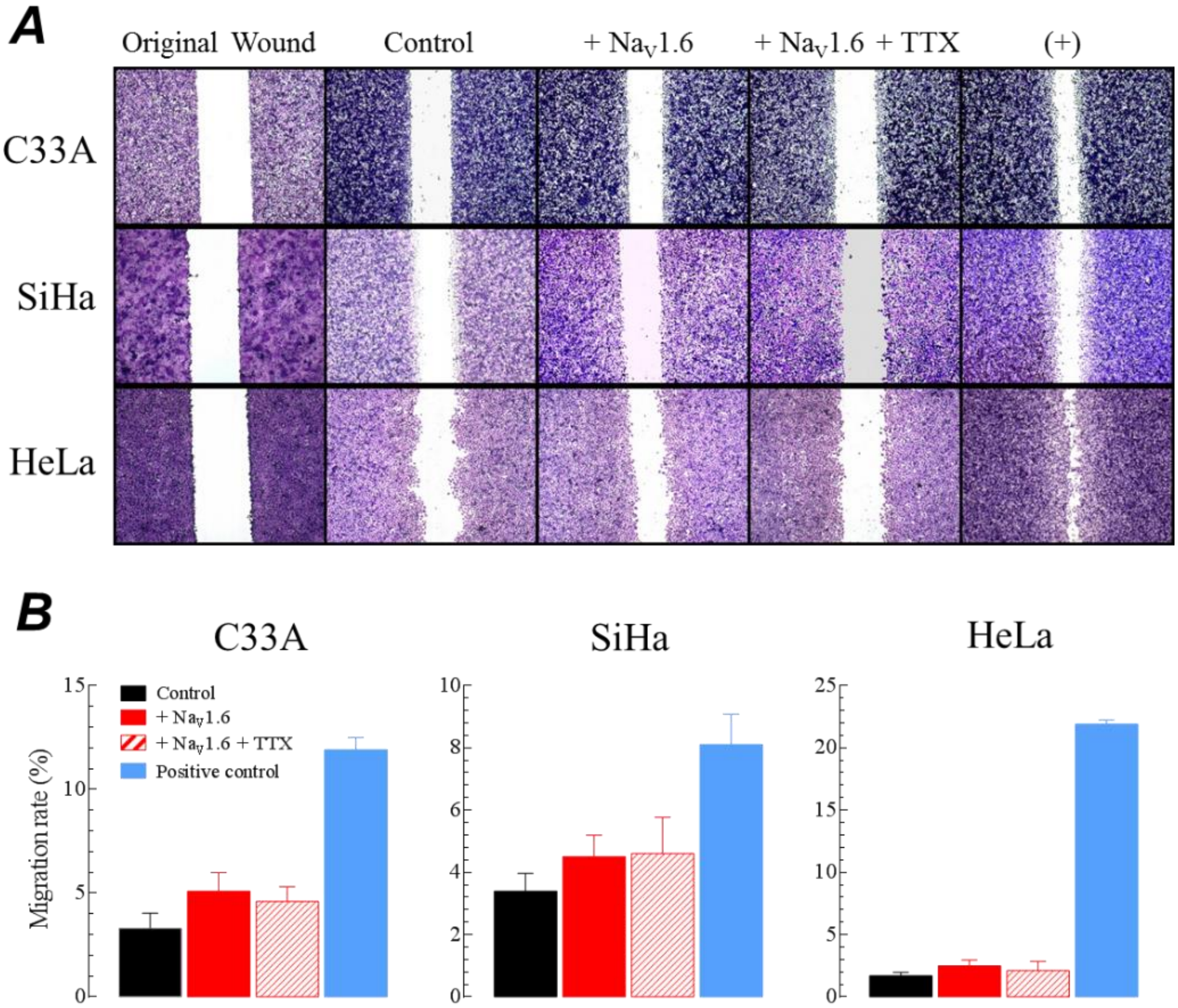

Supplementary Figure 7. Migration of CeCa cell lines is not affected by the heterologous expression of Nav1.6 channels. (**A**) C33A, SiHa and HeLa cells were transfected with the $Na_V1.6$ cDNA, grown in 35 mm dishes and pretreated with 12 μM mitomycin C for 2 h. Cell monolayers were scratch-wounded and incubated in low-serum medium in the absence (+ Nav1.6) or the presence of 1 μM TTX (+ Nav1.6 + TTX). Cell migration into the wounds was evaluated by taking photographs 48 h after wounding. *Original Wound* column represents how the wound looks just after doing it (starting point). *Control* column represents the mock transfection condition. High-serum medium was used as positive control. (**B**) Quantification of wound closure relative to total area of the original wound. Results are given as the mean ± SEM. No significant differences were found among the control cells and those transfected with $Na_V1.6$ channels. For all CeCa cell lines the incubation with high-serum medium (Positive control) resulted in the highest migration rate condition (blue columns).

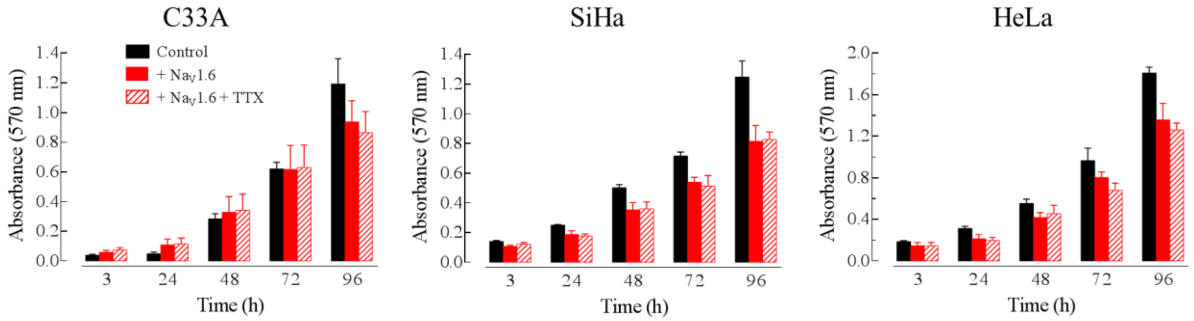

Supplementary Figure 8. Proliferation of CeCa cell lines is not affected by the heterologous expression of Nav1.6 channels. C33A, SiHa and HeLa cells were transfected with Na_V1.6 cDNA, seeded in 48-well plates at cellular density of 5×10^3 cells/well and grown in the absence or the presence of 1 µM TTX. For each experimental point, cell were incubated with MTT (3-(4,5-Dimethylthiazol-2-yl)-2,5-Diphenyltetrazolium Bromide) during 3 h and then absorbance was measured at 570 nm using DMSO as blank. Results are given as the mean ± SEM. The difference between the three experimental conditions was not statistically significant.

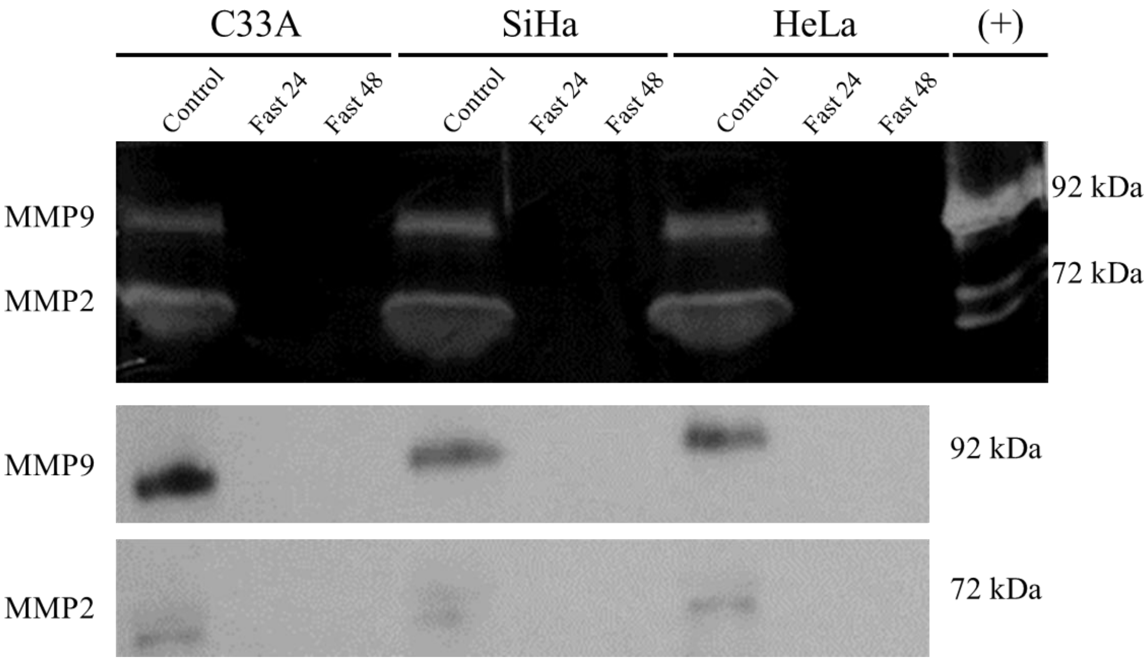

Supplementary Figure 9. Conditioned medium decreases activity of MMP2 and MMP9. Gelatin zymography for control and conditioned medium from cervical cancer cell lines cultures. C33A, SiHa and HeLa cells were grown in complete medium (Control) or in medium without fetal bovine serum (Fast) for 12 and 24 h. Media were recovered from culture plates and concentrated by centrifugation. Activity for gelatinases MMP-2 and MMP-9 was analyzed on equal volumes of each sample by using gelatin-substrate polyacrylamide gel electrophoresis followed by an incubation in activity buffer and a staining with Coomassie blue. Conditioned medium obtained from MCF-7 cells treated with 100 ng/ml phorbol 12, 13-dibutyrate (PDB) for 40 h, was used as positive control. Proteolytic activity was detected as clear bands against a dark background of undigested substrate (*upper panel*). Notice that cervical cancer cells growing in complete medium show a high proteolytic activity of MMP2 and MMP9 proteases, whereas under conditioned media, proteolytic activity was not detected neither 12 h nor 24 h of fasting. Western blot experiments were performed from the same concentrated media analyzed above (*lower panel*). Bands for both, MMP2 and MMP9, were detected in all control media but not in the conditioned ones regardless of the fasting time. The results shown are representative of three independent experiments.

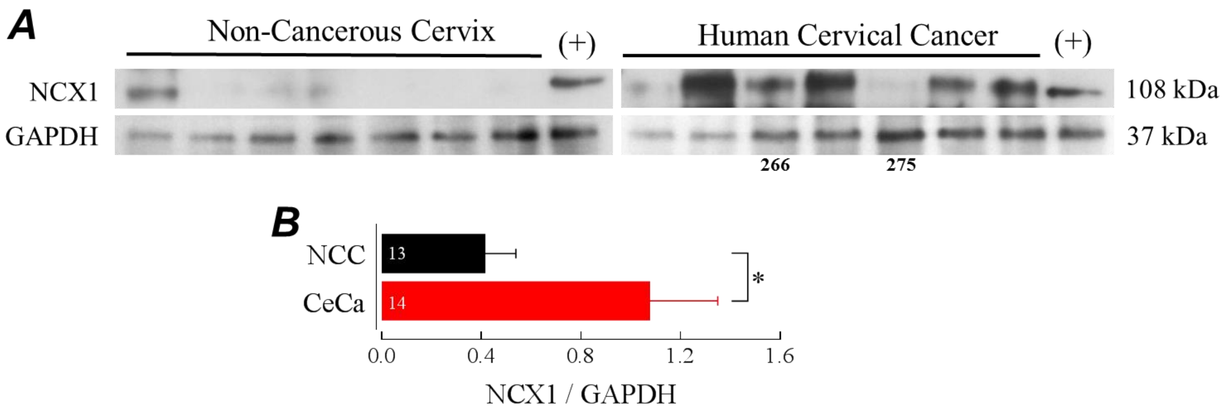

Supplementary Figure 10. Up-regulation of NCX-1 protein in CeCa biopsies. (**A**) Representative western blot for NCX-1 expression in total protein extracts from non-cancerous cervix and human cervical cancer. Total protein extract from mouse brain was used as positive control. (**B**) Evaluation of NCX-1 protein expression. Quantification was made by densitometry analysis of western blot images. Results are given as the amount of NCX-1 protein relative to that of GAPDH in NCC (n = 13) and CeCa (n = 14). Columns are means ± SEM, from two independent experiments. *$P < 0.05$. Samples used in A correspond with those used in Fig. 6C and 6E of the main text.

Supplementary Figure 11. Full scan of western blots, agarose gels and gelatin zymographies shown in figures 1, 3, 4, 6 and 7.

Blots shown in Figure 1C

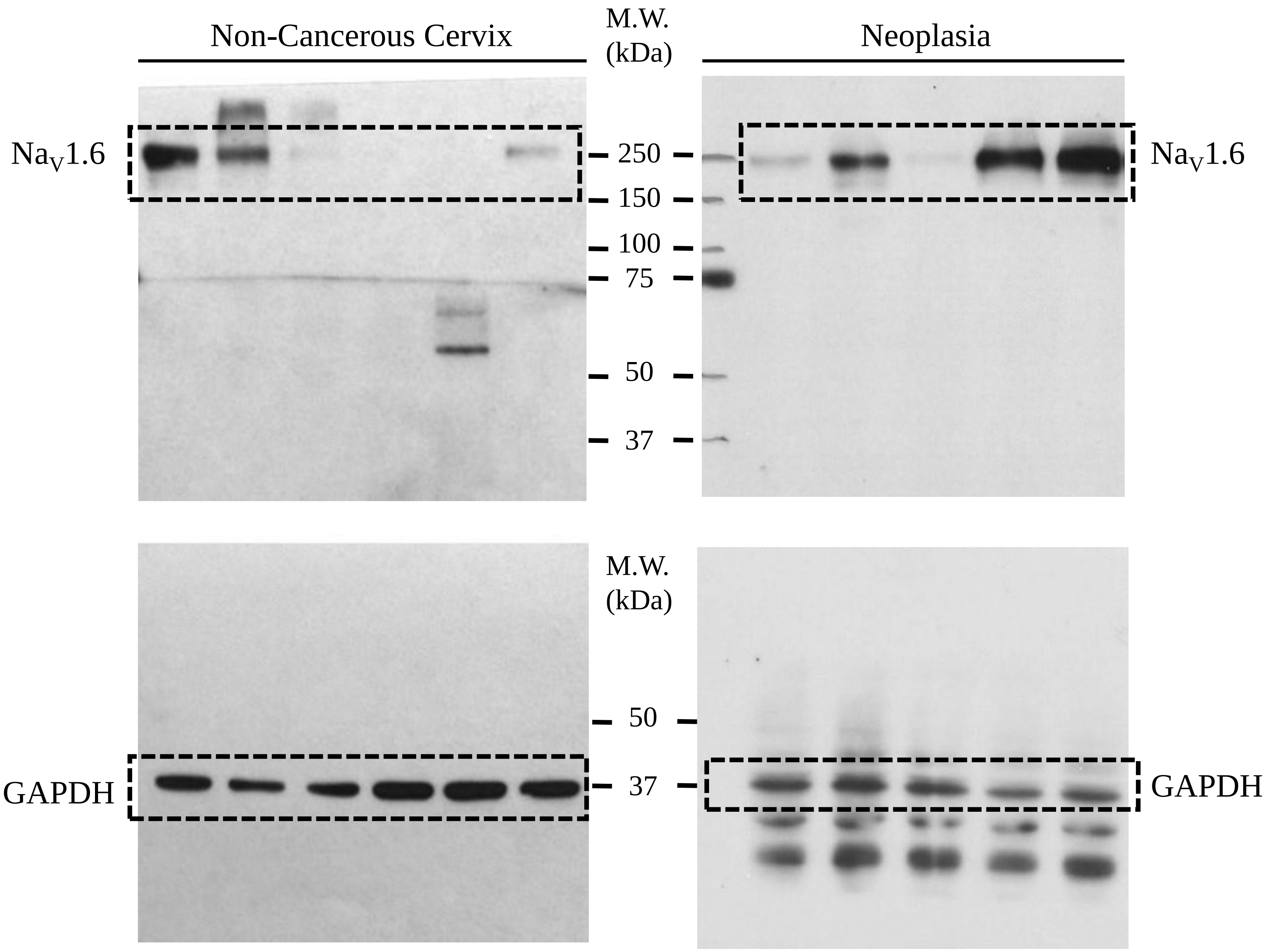

Blots shown in Figure 1C

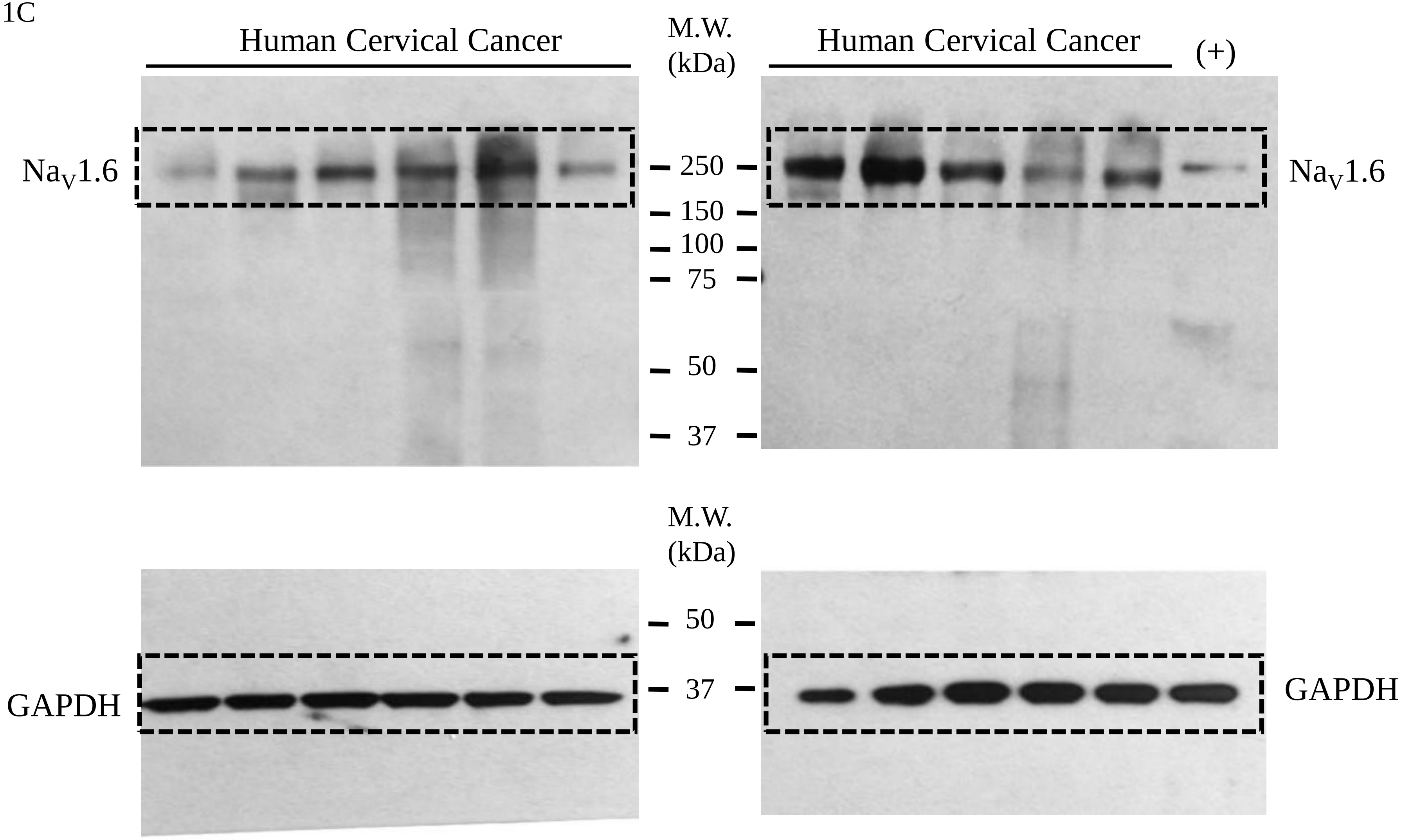

Blots shown in Figure 3B

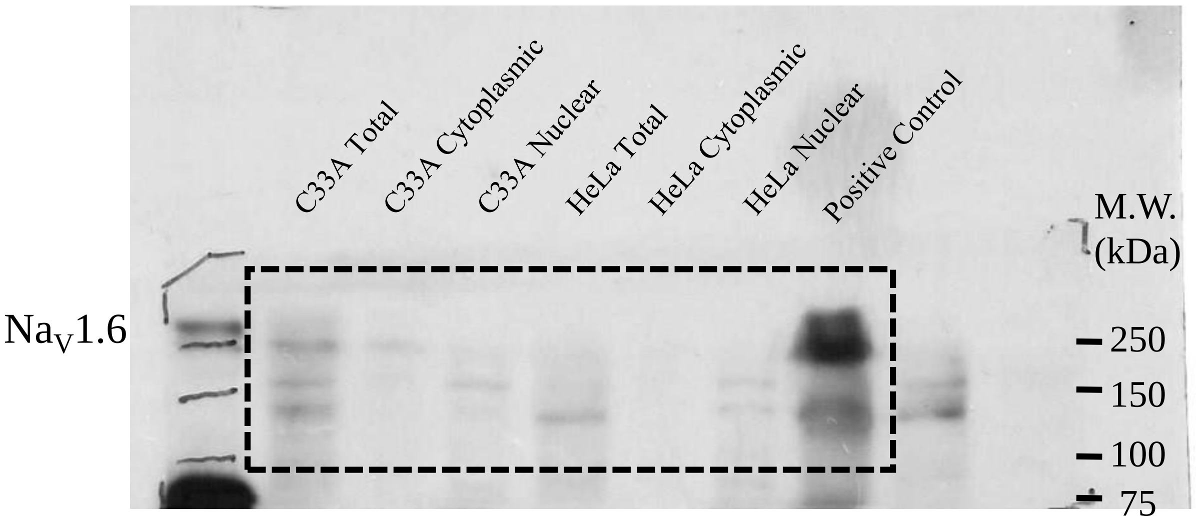

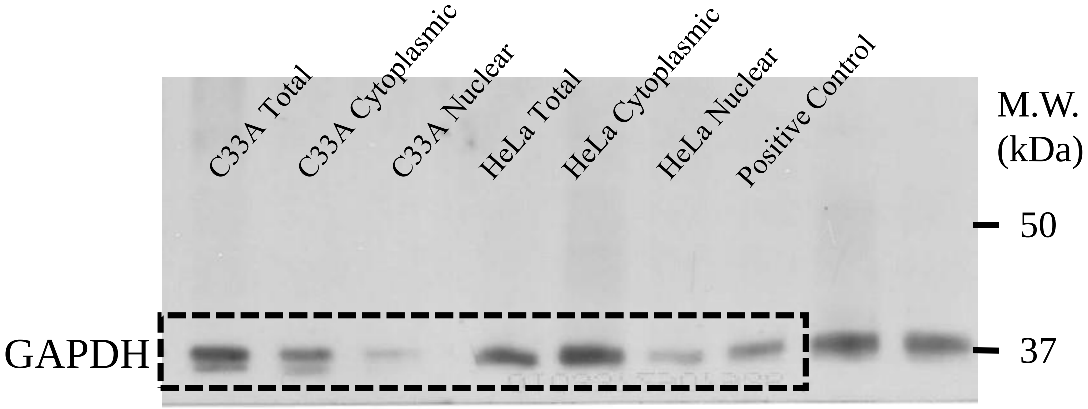

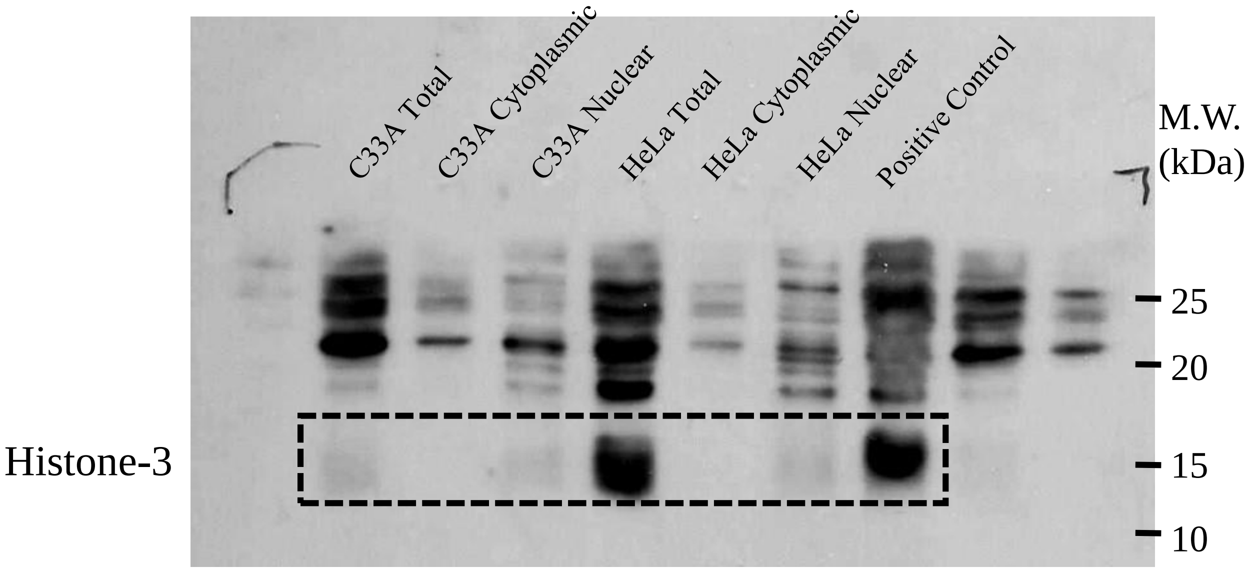

Agarose gels shown in Figure 4B-C

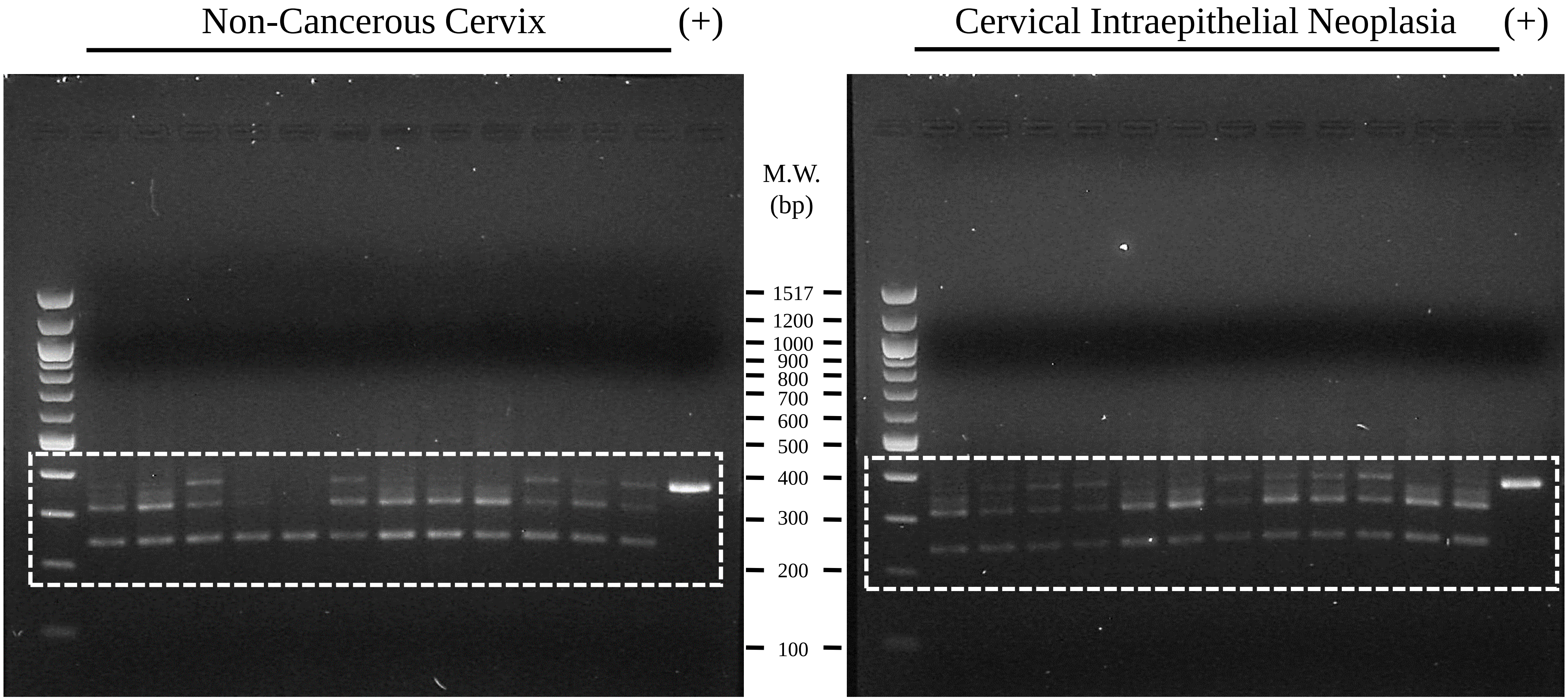

Agarose gels shown in Figure 4D-E

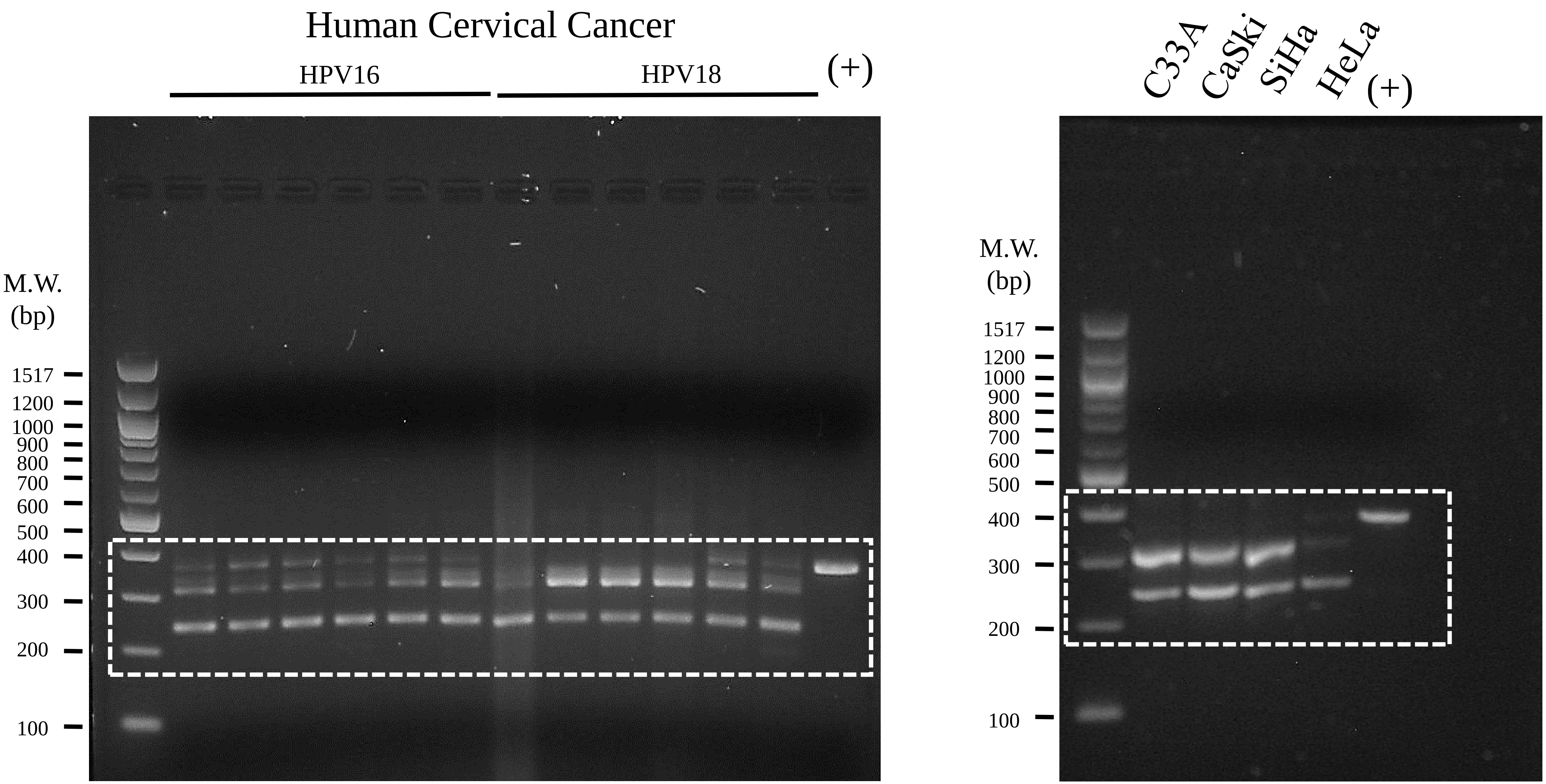

Zymography and blots shown in Figure S8

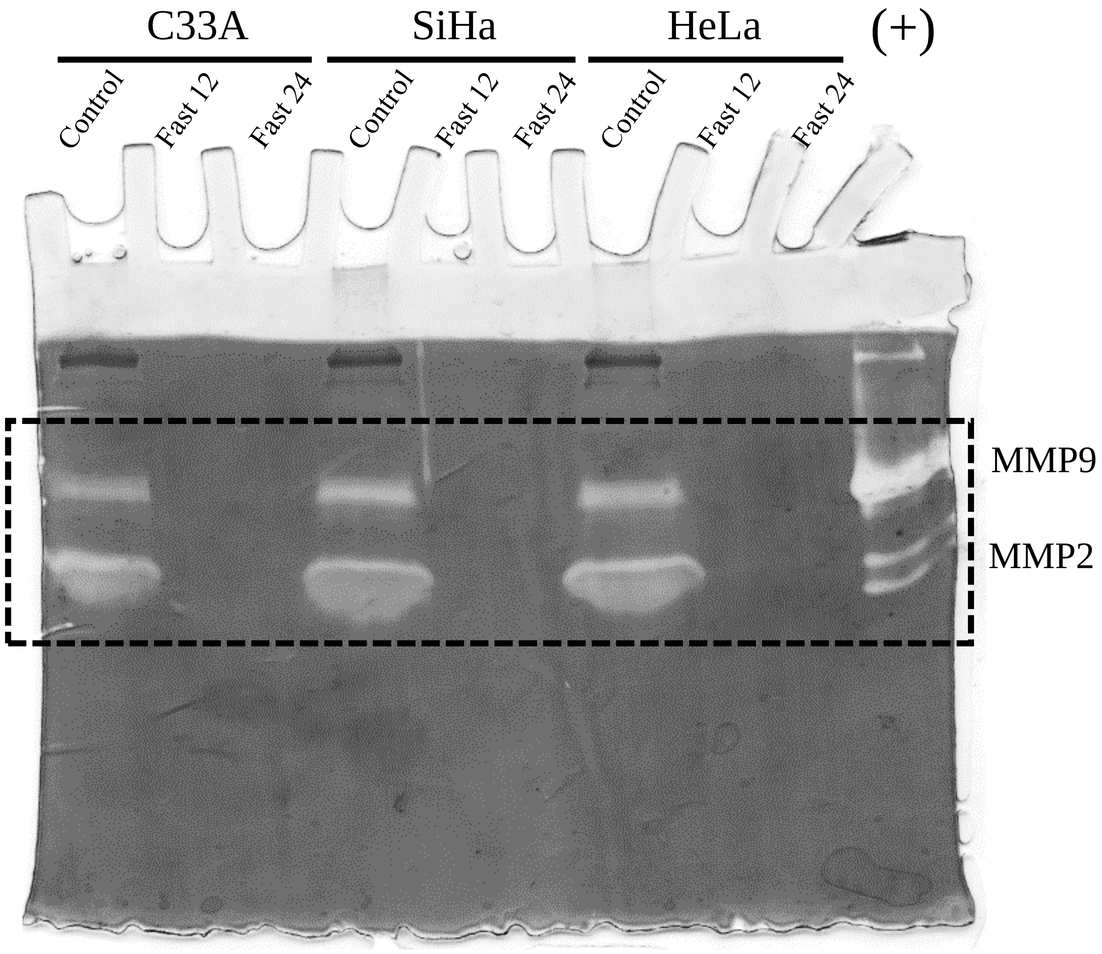

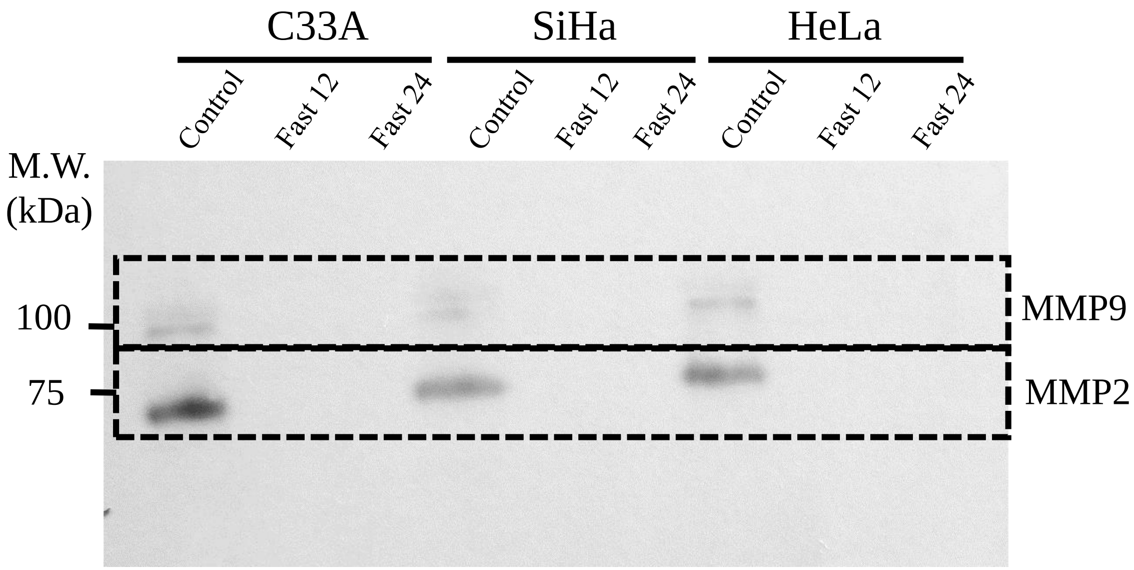

Zymographies and blots shown in Figure 6A

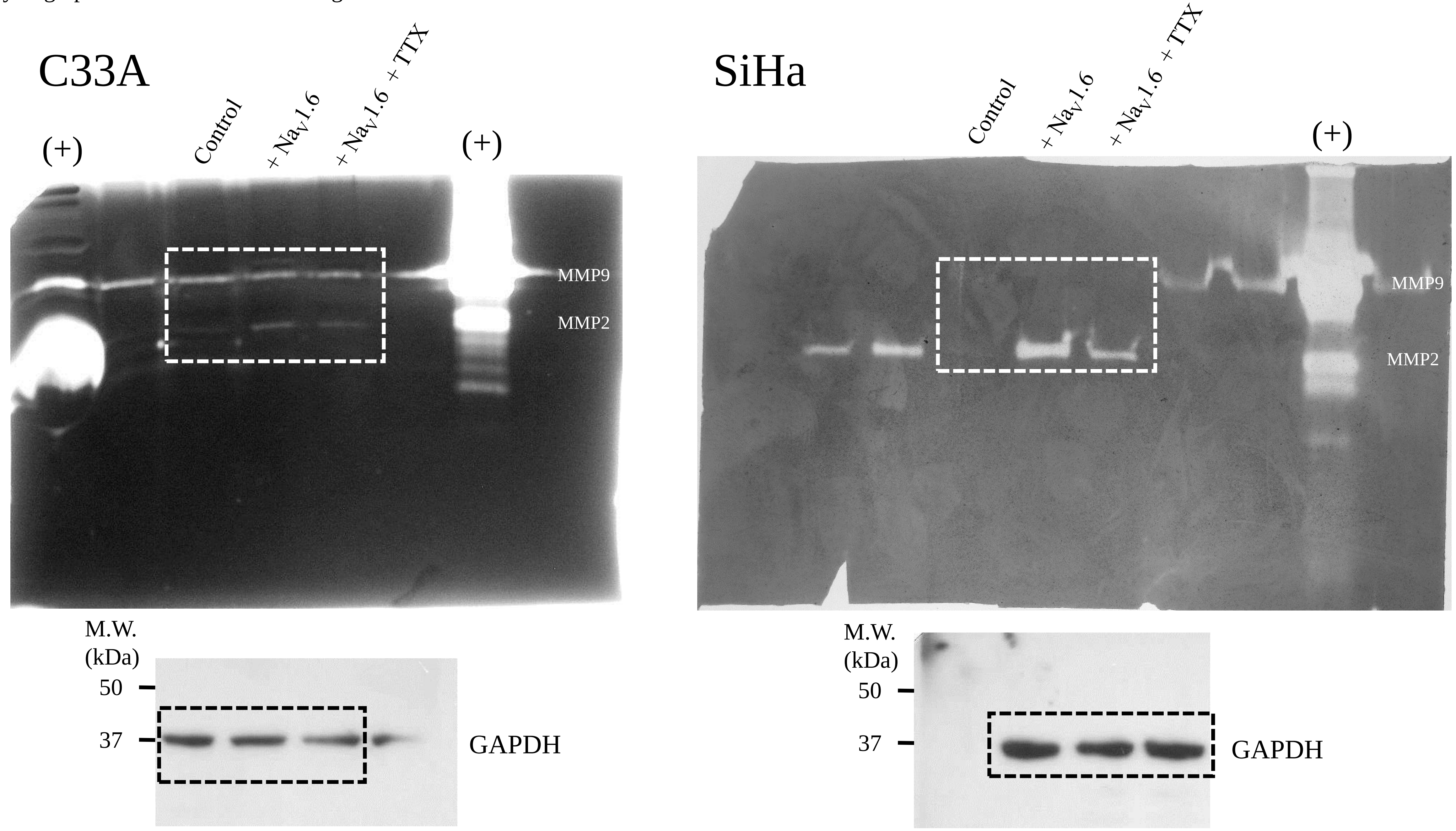

Zymography and blot shown in Figure 6A

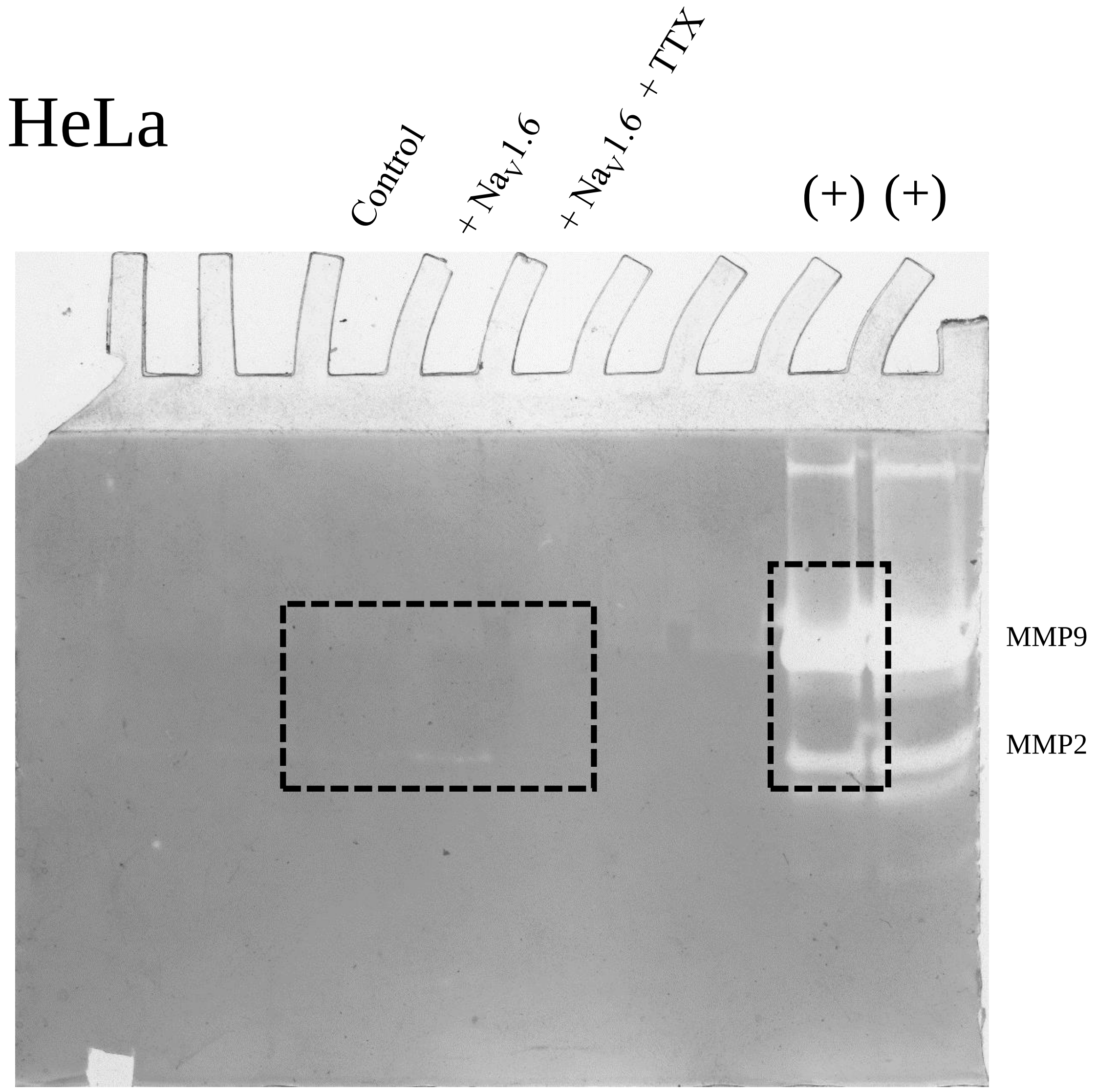

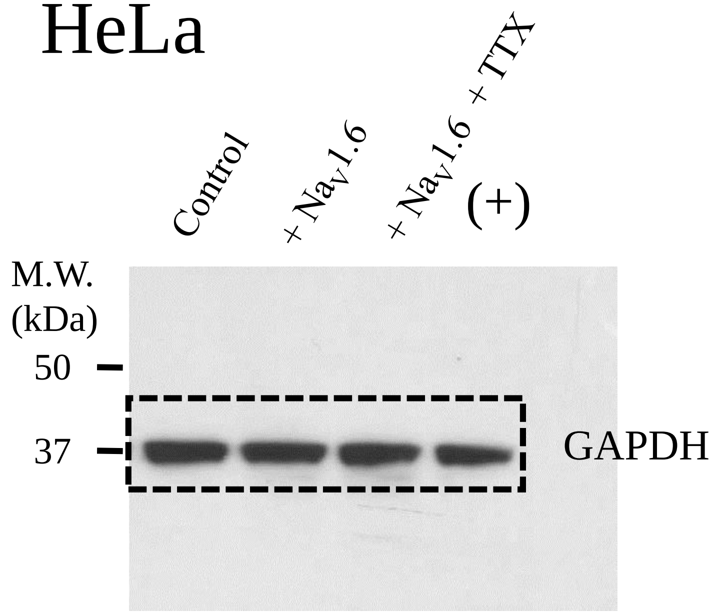

Blots shown in Figure 6C

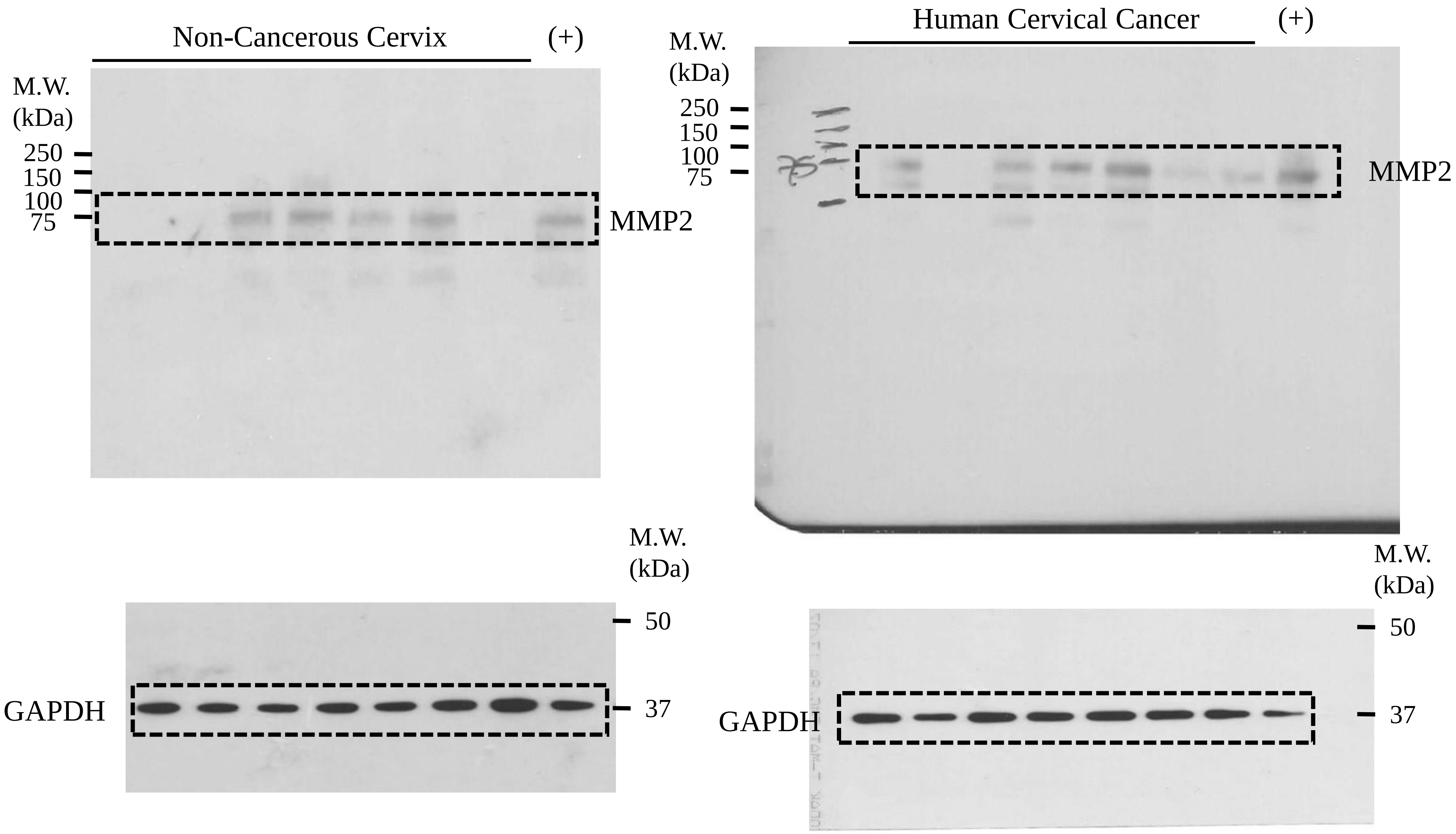

Blots shown in Figure 6E

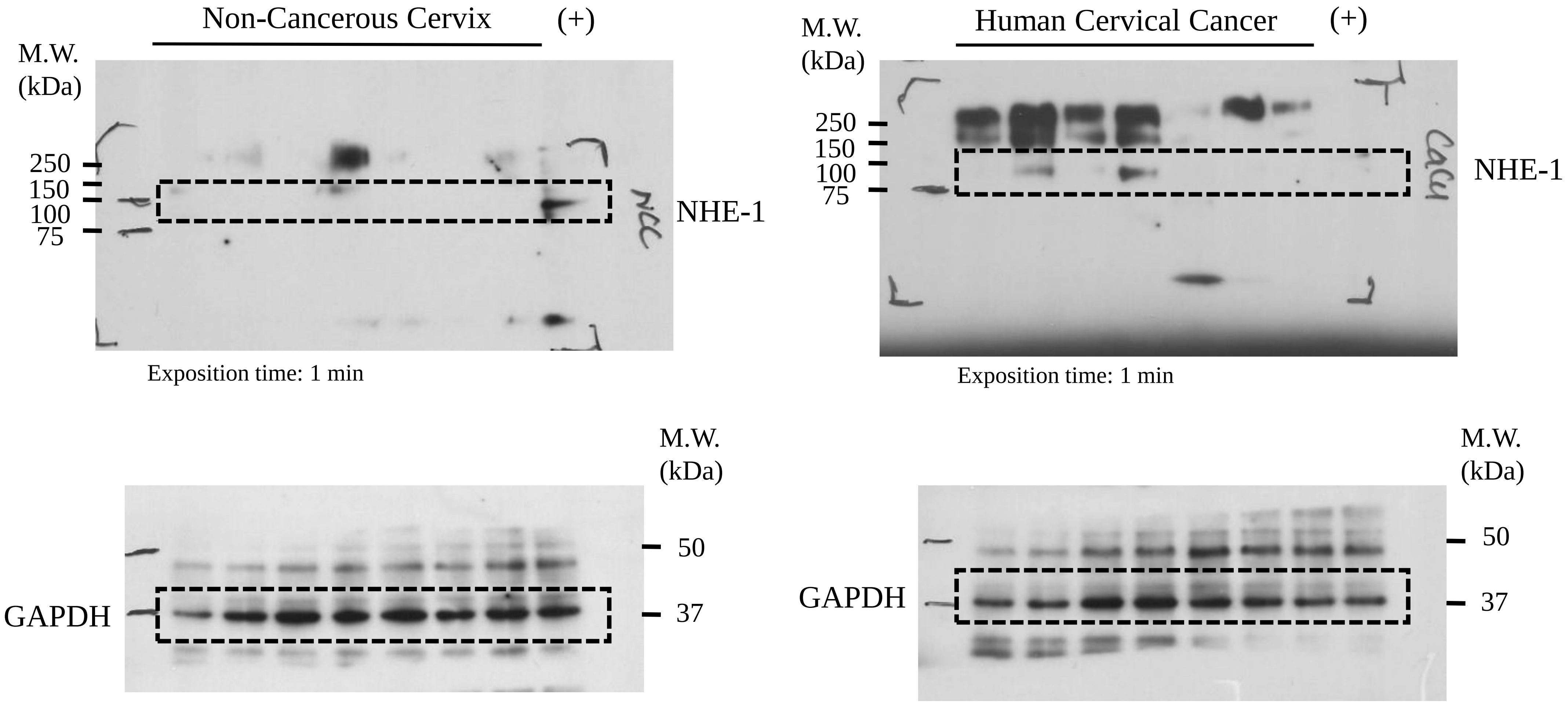

Blots shown in Figure 6E

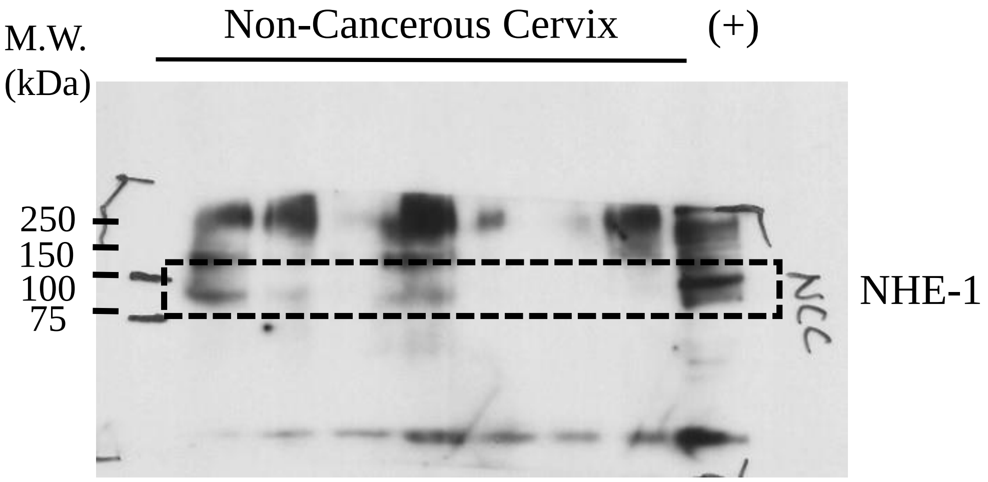

Exposition time: 2 min

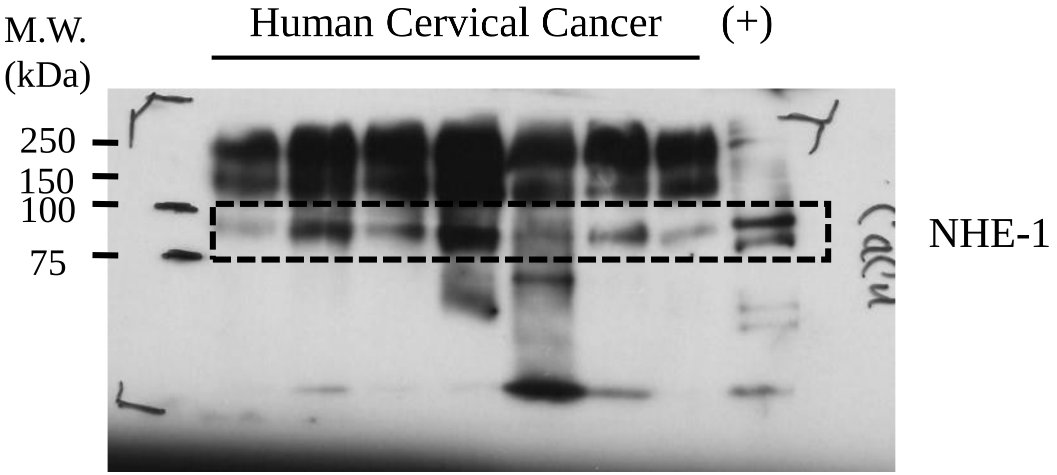

Exposition time: 2 min

Blots shown in Figure 7B

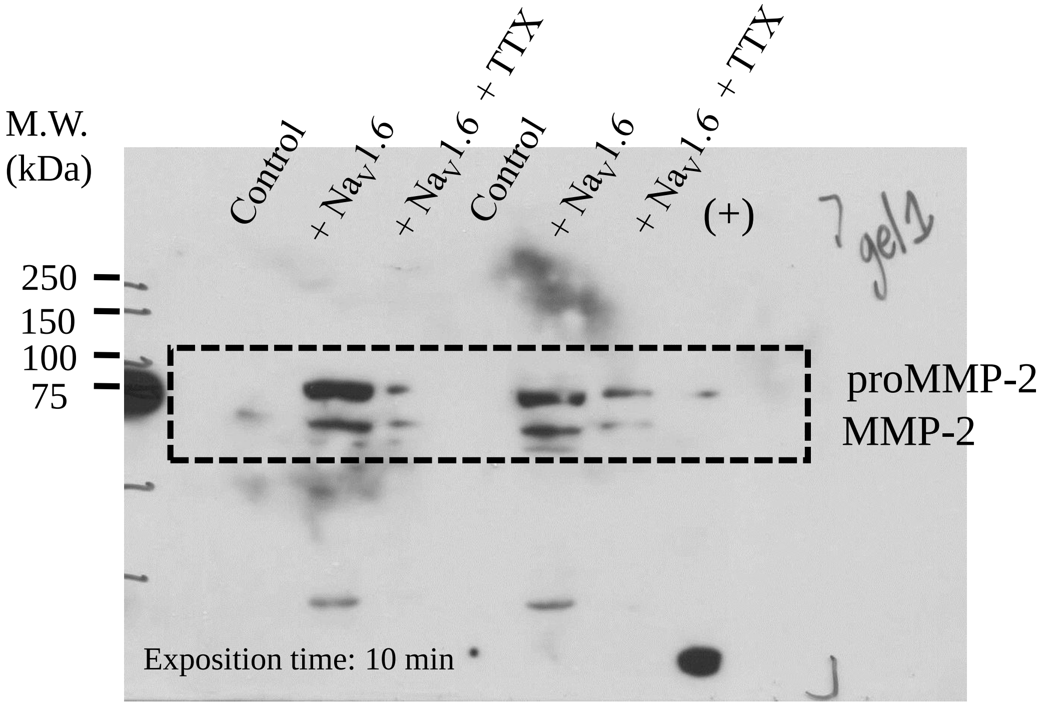

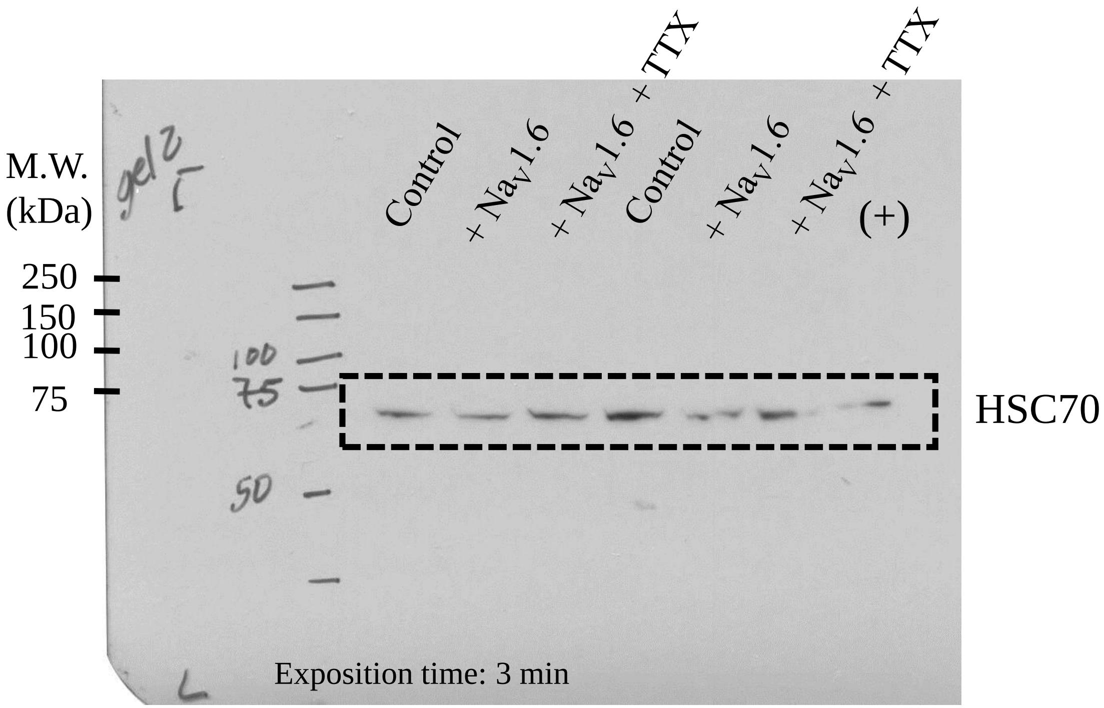

Supplement: Supplementary file 1 — Supplementary Information [file 41598_2018_31364_MOESM1_ESM.pdf]
